# National Institute for Health and Care Excellence (NICE) guidance on monitoring and management of Barrett’s oesophagus and stage I oesophageal adenocarcinoma

**DOI:** 10.1136/gutjnl-2023-331557

**Published:** 2024-03-29

**Authors:** Massimiliano di Pietro, Nigel J Trudgill, Melina Vasileiou, Gaius Longcroft-Wheaton, Alexander W Phillips, James Gossage, Philip V Kaye, Kieran G Foley, Tom Crosby, Sophie Nelson, Helen Griffiths, Muksitur Rahman, Gill Ritchie, Amy Crisp, Stephen Deed, John N Primrose

**Affiliations:** 1 Early Cancer Institute, Department of Oncology, University of Cambridge, Cambridge, UK; 2 Department of Gastroenterology, Sandwell and West Birmingham Hospitals NHS Trust, West Bromwich, UK; 3 Institute of Cancer and Genomic Sciences, University of Birmingham, Birmingham, UK; 4 National Institute for Health and Care Excellence, London, UK; 5 Department of Gastroenterology, Portsmouth Hospitals NHS Trust, Portsmouth, UK; 6 Department of Pharmacy and Biomedical Sciences, University of Portsmouth, Portsmouth, UK; 7 Department of Surgery, Newcastle Upon Tyne Hospitals NHS Foundation Trust, Newcastle Upon Tyne, UK; 8 Department of Gastrointestinal Surgery, St Thomas' Hospital, London, UK; 9 Department of Histopathology, Nottingham University Hospitals NHS Trust, Nottingham, UK; 10 Division of Cancer and Genetics, Cardiff University, Cardiff, Cardiff, UK; 11 Department of Clinical Oncology, Velindre University NHS Trust, Cardiff, UK; 12 Kenmore Medical Centre, Manchester University NHS Foundation Trust, Manchester, UK; 13 Powys Teaching Health Board, Bronllys, UK; 14 Department of Surgery, University of Southampton, Southampton, UK

**Keywords:** BARRETT'S OESOPHAGUS, OESOPHAGEAL CANCER, PROTON PUMP INHIBITION, ANTI-REFLUX SURGERY, SURVEILLANCE

## Abstract

Barrett’s oesophagus is the only known precursor to oesophageal adenocarcinoma, a cancer with very poor prognosis. The main risk factors for Barrett’s oesophagus are a history of gastro-oesophageal acid reflux symptoms and obesity. Men, smokers and those with a family history are also at increased risk. Progression from Barrett’s oesophagus to cancer occurs via an intermediate stage, known as dysplasia. However, dysplasia and early cancer usually develop without any clinical signs, often in individuals whose symptoms are well controlled by acid suppressant medications; therefore, endoscopic surveillance is recommended to allow for early diagnosis and timely clinical intervention. Individuals with Barrett’s oesophagus need to be fully informed about the implications of this diagnosis and the benefits and risks of monitoring strategies. Pharmacological treatments are recommended for control of symptoms, but not for chemoprevention. Dysplasia and stage 1 oesophageal adenocarcinoma have excellent prognoses, since they can be cured with endoscopic or surgical therapies. Endoscopic resection is the most accurate staging technique for early Barrett’s-related oesophageal adenocarcinoma. Endoscopic ablation is effective and indicated to eradicate Barrett’s oesophagus in patients with dysplasia. Future research should focus on improved accuracy for dysplasia detection via new technologies and providing more robust evidence to support pathways for follow-up and treatment.

## Executive summary

Barrett’s oesophagus is defined as the adaptive metaplastic change of the oesophageal mucosa from the native multilayered squamous epithelium to a columnar-type epithelium, characteristically with intestinal-type differentiation.^1^ This metaplastic change typically occurs in response to injury of the oesophageal mucosa due to gastro-oesophageal acid reflux. The reasons why only certain individuals with reflux oesophagitis develop Barrett’s oesophagus are not fully understood, but based on available evidence it is plausible to hypothesise that this occurs within a predisposing genetic background, which is estimated to account for 35% of the disease heritability.^2 3^ The molecular and cellular mechanisms leading to the development of Barrett’s oesophagus are also debated. Several hypotheses have been proposed (including transdifferentiation from squamous to columnar cell type, origin from submucosal glands and circulating stem cells), but recent evidence from mouse models and human tissues indicates that the activation of progenitor cells located at the gastro-oesophageal junction upon injury leads to expansion of cells with columnar phenotype and colonisation of the lower oesophagus by the metaplastic epithelium.^4 5^

The usual gold standard for diagnosis of Barrett’s oesophagus is endoscopy, which reveals the extent of the metaplastic condition and allows for histopathological assessment of tissue biopsy samples. The criteria for a diagnosis of Barrett’s oesophagus have been somewhat controversial in the recent past. It is important to consider that the elements in support of this diagnosis should align with the clinical significance of the disease, which essentially revolves around the cancer predisposition and the opportunities to prevent neoplastic transformation in individuals who might be a higher cancer risk compared with the general population.

There have been two areas of intense discussion about the diagnosis of Barrett’s oesophagus. The first relates to the cell phenotype required for the pathological diagnosis and the second relates to the minimum extent of oesophageal involvement by the metaplastic epithelium to support an endoscopic diagnosis. Some specialist societies require the presence of intestinal metaplasia for a diagnosis of Barrett’s oesophagus.^6 7^ The British Society of Gastroenterology has historically disagreed, recognising the possibility of a diagnosis of Barrett’s oesophagus with gastric metaplasia only.^8^ It is well known that from a histopathologic perspective that Barrett’s oesophagus is a mixture of different phenotypes, where the intestinal type cells can be more or less predominant.^1 8^ Although the NICE guideline committee did not disagree with the possibility of a non-intestinalised Barrett’s oesophagus, it did recognise the evidence that individuals with suspected Barrett’s oesophagus and intestinal metaplasia in biopsy specimens have higher cancer risk than those with gastric-type epithelium only.^8 9^ As for the length of metaplastic epithelium for an endoscopic diagnosis, retrospective evidence suggests that segments of metaplastic epithelium shorter than 1 cm are associated with a negligible cancer risk.^10^ Diagnostic criteria for Barrett’s oesophagus are outside the scope of this guideline, but the committee were aware that the British Society of Gastroenterology recommends that a length of at least 1 cm is required for an endoscopic diagnosis of Barrett’s oesophagus^8^ and this is also standard clinical practice outside the UK.^6 7^

Once a diagnosis of Barrett’s oesophagus is made based on the synthesis of endoscopic and histopathological data, it is important to ensure that this diagnosis is discussed with the patient and, if appropriate, a plan is agreed to monitor this and minimise the consequences of incidental cancer progression. The NICE guideline committee has systematically analysed the evidence on pharmacological, endoscopic and surgical interventions to prevent, monitor and treat individuals with Barrett’s oesophagus and related early neoplasia and produced the following series of recommendations to support healthcare professionals managing individuals with this condition. The principal patient group are those found to have Barrett’s oesophagus with or without dysplasia and those with early oesophageal adenocarcinoma limited to the mucosal or submucosal layers. The target users include gastroenterologists, GI surgeons, pathologists, endoscopists, specialist nurses and general practitioners. Finally, population-level screening was outside the scope of the guideline. These recommendations are listed below:

### 1.1 Information and support

1.1.1 Offer a clinical consultation to people with newly diagnosed Barrett’s oesophagus to discuss the risk of cancer, endoscopic surveillance plans and symptom control.

1.1.2 Give the person verbal and written information about their diagnosis, available treatments and patient support groups. Give them time to consider this information when making decisions about their care.

1.1.3 After each surveillance procedure, provide the person with an endoscopy report that includes a lay summary of the findings and a reference to ongoing symptom control.

1.1.4 Follow the recommendations on communication and information in the NICE guidelines on patient experience in adult NHS services and shared decision-making.

### 1.2 Pharmacological interventions

#### Symptom control

1.2.1 Follow the recommendations on interventions for gastro-oesophageal reflux disease (GORD) in the NICE guideline on gastro-oesophageal reflux disease and dyspepsia in adults.

#### Preventing disease progression

1.2.2 Do not offer aspirin to people with Barrett’s oesophagus to prevent progression to oesophageal dysplasia and cancer.

### 1.3 Endoscopic surveillance

1.3.1 Discuss the benefits and risks of endoscopic surveillance with the person diagnosed with Barrett’s oesophagus.

1.3.2 Offer high-resolution white light endoscopy with Seattle biopsy protocol for surveillance of Barrett’s oesophagus. Take into account the health of the person and ensure the benefits of surveillance outweigh the risks.

#### Frequency of endoscopic surveillance

1.3.3 Offer high resolution white light endoscopic surveillance with Seattle protocol biopsies:

- every 2 to 3 years for people with long-segment (≥3 cm) Barrett’s oesophagus

- every 3 to 5 years to people with short-segment (<3 cm) Barrett’s oesophagus with intestinal metaplasia.

1.3.4 Assess a person’s risk of cancer based on their age, sex, family history of oesophageal cancer and smoking history, and tailor the frequency of endoscopic surveillance accordingly.

1.3.5 Do not offer endoscopic surveillance to people with short-segment (<3 cm) Barrett’s oesophagus without intestinal metaplasia provided the diagnosis has been confirmed by two endoscopies.

### 1.4 Staging for suspected stage 1 oesophageal adenocarcinoma

1.4.1 Offer endoscopic resection for staging, to people with suspected stage 1 oesophageal adenocarcinoma.

1.4.2 Do not use CT before endoscopic resection for staging suspected T1 oesophageal adenocarcinoma.

1.4.3 Do not use endoscopic ultrasonography (EUS) before endoscopic resection for staging suspected T1a oesophageal adenocarcinoma.

1.4.4 Consider EUS for nodal staging, for people with suspected T1b oesophageal adenocarcinoma based on endoscopic appearances or diagnosed with T1b oesophageal adenocarcinoma based on histological examination of endoscopic resection specimens.

### 1.5 Managing Barrett’s oesophagus with dysplasia

1.5.1 Offer endoscopic resection of visible oesophageal lesions as first-line treatment to people with high-grade dysplasia.

1.5.2 Offer endoscopic ablation of any residual Barrett’s oesophagus to people with high-grade dysplasia after treatment with endoscopic resection.

1.5.3 Offer radiofrequency ablation to people with low-grade oesophageal dysplasia diagnosed from biopsy samples taken at two separate endoscopies. Two gastrointestinal pathologists should confirm the histological diagnosis.

1.5.4 Consider endoscopic surveillance at 6 monthly intervals with dose optimisation of acid-suppressant medication for people diagnosed with indefinite dysplasia of the oesophagus.

1.5.5 Offer endoscopic follow-up to people who have received endoscopic treatment for Barrett’s oesophagus with dysplasia.

1.5.6 Follow the NICE interventional procedures guidance on endoscopic radiofrequency ablation for Barrett’ s oesophagus with low-grade dysplasia or no dysplasia and epithelial radiofrequency ablation for Barrett’ s oesophagus. https://www.nice.org.uk/guidance/ipg496

### 1.6 Managing stage 1 oesophageal adenocarcinoma

1.6.1 Offer a clinical consultation to people with stage 1 oesophageal adenocarcinoma to discuss and evaluate the suitability of treatment options, including endoscopic resection or oesophagectomy.

1.6.2 Offer endoscopic resection as first-line treatment to people with T1a oesophageal adenocarcinoma.

1.6.3 Offer endoscopic ablation of any residual Barrett’s oesophagus to people with T1a oesophageal adenocarcinoma after treatment with endoscopic resection.

1.6.4 Offer endoscopic follow-up to people who have received endoscopic treatment for stage 1 oesophageal adenocarcinoma.

1.6.5 Offer oesophagectomy to people with T1b oesophageal adenocarcinoma who are fit for surgery and at high risk of cancer progression. For example, where there is:

- incomplete endoscopic resection

- evidence of lymphovascular invasion or deep submucosal invasion (more than 500 μm) on histological examination of endoscopic resection specimens.

### 1.7 Non-surgical treatment for T1b oesophageal adenocarcinoma

1.7.1 Consider radiotherapy (alone or in combination with chemotherapy) for people with T1b oesophageal adenocarcinoma at high risk of cancer progression (for example, incomplete endoscopic resection, or evidence of lymphovascular invasion or deep submucosal invasion (>500 μm) on histological examination of endoscopic resection specimens) and who are unfit for oesophagectomy.

1.7.2 Offer endoscopic follow-up to people who have received radiotherapy for T1b oesophageal adenocarcinoma.

### 1.8 Anti-reflux surgery

1.8.1 Do not offer anti-reflux surgery to people with Barrett’s oesophagus to prevent progression to dysplasia or cancer.

1.8.2 Follow the recommendations on laparoscopic fundoplication for gastro-oesophageal reflux disease in the NICE guideline on gastro-oesophageal reflux disease and dyspepsia in adults.

## 2. Objective

The objective of this guideline was to provide evidence-based recommendations for the management of patients with Barrett’s oesophagus and stage 1 oesophageal adenocarcinoma. This included recommendations for information and support, pharmacological treatments, use of endoscopic, non-endoscopic and radiological tests for monitoring and staging of Barrett’s and suspected stage 1 adenocarcinoma, and indications for endoscopic and surgical techniques to treat Barrett’s and related neoplasia. This guideline did not review any evidence related to the diagnostic criteria for Barrett’s oesophagus and management of adenocarcinoma stage 2 or higher.

## 3. NICE guideline processes and methods

The guideline was developed using the methods described in the NICE guidelines manual.^11^ NICE develops evidence-based guidelines based on the best available clinical and health economic evidence.

A scope was developed detailing the topic areas and clinical questions to be addressed within the guideline. A consultation to seek the views of stakeholders was held, and the scope was amended based on the stakeholder comments received.

A committee to develop the guideline was appointed through an open application process, consisting of clinicians with experience in management of Barrett’s oesophagus and stage 1 oesophageal adenocarcinoma, as well as lay members. The committee included gastroenterologists, upper GI surgeons, radiologist, pathologist, oncologist, nurse endoscopist, general practitioners (GP), dietician and lay members.

### Developing clinical questions and literature searches

Fourteen review questions were developed based on the key areas of the guideline scope (online supplementary table 1). A review protocol was developed for each question using a framework defining the different aspects making up the review question. The PICO framework was used to define: the population, the intervention, the comparator and the outcomes for intervention reviews. The population, index test, reference standard and target condition framework was used for diagnostic test accuracy reviews, and population, setting and context for qualitative reviews. The frameworks guided the systematic literature searches undertaken for each review question, to identify relevant published clinical and health economic evidence and inform the recommendations made by the guideline committee.

Electronic databases were searched using relevant medical subject headings, free-text terms derived from the protocol frameworks and, where appropriate, study-type filters to prioritise the inclusion of evidence of the highest available quality. For intervention reviews, randomised controlled trials (RCTs) considered the most appropriate type of study to assess the effectiveness of interventions, were searched for using Medline, Embase, The Cochrane Library and Epistemonikos electronic databases. Where no evidence from RCTs was available, non-randomised studies were subsequently searched for and included as appropriate. For diagnostic accuracy reviews, a search was made for observational studies. Qualitative studies, questionnaires and surveys were searched for qualitative reviews using Medline, Embase, Current Nursing and Allied Health, Literature (CINAHL) and PsycINFO electronic databases.

### Clinical evidence synthesis and grading of evidence

Evidence identified was assessed against the prespecified inclusion/exclusion criteria of each review protocol. Key information from studies selected for inclusion were tabulated in evidence tables, listing the study design, details of the population, intervention/index text, comparison or reference standard and results. Evidence tables also included critical appraisal ratings for each study, derived using the preferred study design checklist as specified in the NICE guidelines manual.^11^

Quantitative outcome data were combined and meta-analysed where appropriate, or reported individually where variability in the studies did not allow pooling of data. These were then summarised in Grading of Recommendations Assessment, Development and Evaluation (GRADE) evidence profiles.^12^ Meta-analyses were conducted using Cochrane Review Manager version 5.3 (ReVMan5) software.

Qualitative data were extracted in a narrative format and synthesised across studies using thematic analysis and presented as summary statements in GRADE CERQual (Confidence in Evidence from Reviews of Qualitative research) tables. Quantitative data, questionnaires and surveys related to the qualitative data were extracted in narrative format and included in the qualitative synthesis to help illustrate the themes emerging from the qualitative studies.

The quality of the evidence from quantitative studies was assessed using the GRADEpro software developed by the international GRADE working group. This is evaluated on an outcome level, taking into account individual study quality and meta-analysis results, based on the quality elements outlined in table 1.

**Table 1:** Assessing the quality of the evidence

Quality element	Description
Risk of bias	Limitations in the study design and implementation might bias the estimates of the treatment effect. Major limitations in studies decrease the confidence in the estimate of the effect. Examples of such limitations are selection bias (often due to poor allocation concealment), performance and detection bias (often due to a lack of blinding of the patient, healthcare professional or assessor) and attrition bias (due to missing data causing systematic bias in the analysis).
Indirectness	Indirectness refers to differences in study population, intervention, comparator and outcomes between the available evidence and the review question.
Inconsistency	Inconsistency refers to an unexplained heterogeneity of effect estimates between studies in the same meta-analysis.
Imprecision	Results are imprecise when studies include relatively few patients and few events (or highly variable measures) and thus have wide confidence intervals around the estimate of the effect relative to clinically important thresholds. 95% confidence intervals denote the possible range of locations of the true population effect at a 95% probability, and so wide confidence intervals may denote a result that is consistent with conflicting interpretations (for example, a result may be consistent with both clinical benefit AND clinical harm) and thus be imprecise.
Publication bias	Publication bias is a systematic underestimate or overestimate of the underlying beneficial or harmful effect due to the selective publication of studies. A closely related phenomenon is where some papers fail to report an outcome that is inconclusive, thus leading to an overestimate of the effectiveness of that outcome.
Other issues	Sometimes randomisation might not adequately lead to group equivalence of confounders, and if so, this might lead to bias, which should be taken into account. Potential conflicts of interest, often caused by excessive pharmaceutical company involvement in the publication of a study, should also be noted.

Following the appraisal of each outcome, an overall quality grade was derived, and evidence was graded as either high, moderate, low or very low quality (table 2).

**Table 2:** GRADE quality ratings

Level	Description
High	Further research is very unlikely to change our confidence in the estimate of effect
Moderate	Further research is likely to have an important impact on our confidence in the estimate of effect and may change the estimate
Low	Further research is very likely to have an important impact on our confidence in the estimate of effect and is likely to change the estimate
Very low	Any estimate of effect is very uncertain

A similar approach was followed for qualitative evidence, where the GRADE-CERQual was used to assess the body of evidence synthesised under each review theme through a confidence rating representing the extent to which a review finding is an accurate representation of the phenomenon of interest. The level of confidence was categorised as high, moderate, low and very low that the review finding is a reasonable representation of the phenomenon of interest.

### Health economic evidence methods

The committee was required to make decisions based on the best available evidence of both clinical effectiveness and cost effectiveness. Health economic evidence was therefore sought relating to the review questions.

Health economists undertook a systematic review of the published economic literature. They began by identifying potentially relevant studies for each review question from the health economic search results by reviewing titles and abstracts. Full papers were obtained, then reviewed against prespecified inclusion and exclusion criteria.^11^ The health economists then critically appraised relevant studies for applicability and methodological quality using economic evaluations checklists.^11^

Key information about the studies’ methods and results was extracted into health economic evidence reviews. NICE health economic evidence profile tables were used to summarise cost and cost-effectiveness estimates for the included health economic studies in each evidence review report. The health economic evidence profile showed an assessment of applicability and methodological quality for each economic study, with footnotes indicating the reasons for the assessment. A summary of costs and cost-effectiveness estimates for the base-case analysis, as well as information of the assessment of uncertainty in the analysis were also reported.

As well as reviewing the published health economic literature for each review question, areas for new analysis were agreed by the committee after formation of the review questions and consideration of the existing health economic evidence. The committee identified three review questions as high priority areas for original health economic modelling. However, the original cost-effectiveness analysis was not feasible in each area due to the lack of robust clinical evidence.

### Developing recommendations

Decisions on whether a recommendation could be made were based on the committee’s interpretation of the available evidence, taking into account the balance of benefits, harms and costs between different courses of action. The net clinical benefit over harm (clinical effectiveness) was considered, focusing on the magnitude of the effect (or clinical importance), quality of evidence and amount of evidence available. The assessment of net clinical benefit was moderated by the importance placed on the outcomes, and the confidence the committee had in the evidence (evidence quality). The committee also assessed whether the clinical effectiveness justified any differences in costs between the alternative interventions.

For many questions the clinical and health economic evidence was of poor quality, or absent. Here, the committee debated whether a recommendation could be made based on its expert opinion. Considerations for making consensus-based recommendations included the balance between potential harms and benefits, the economic costs compared with the economic benefits, current practices, recommendations made in other relevant guidelines, patient preferences and equality issues.

For reviews where good evidence was lacking, the committee considered making recommendations for future research.

## Consultation

The draft guideline was published on the NICE website for consultation with stakeholders as part of the quality assurance and peer review of the document. After consultation the committee discussed the comments received from stakeholders and considered any changes needed to the guideline, and agreed the final wording of the recommendations.

### Information and support

Six studies (four qualitative studies and two questionnaire studies reporting quantitative data) were included in the review about the information and support needs of people with Barrett’s oesophagus^13–18^ (online supplemental table 2).

10.1136/gutjnl-2023-331557.supp1 Supplementary data

Qualitative evidence highlighted knowledge gaps and uncertainties at the time of diagnosis of Barrett’s oesophagus. The committee agreed a clinical consultation should be offered following diagnosis to provide information and support on the risk of progression to cancer and symptom control, and general information about endoscopic surveillance.

Providing information both verbally and in written form is helpful as information can be difficult to grasp at a single consultation, and written information will enable people to revisit the information when needed. This should include general information about the diagnosis of Barrett’s oesophagus, available treatments and any patient support groups.

The use of complex medical terminology limits people’s ability to understand information. The committee agreed it is important that each endoscopy report includes a lay summary of the findings and that this is given to the person.

The recommendations are in line with current practice and therefore are unlikely to have a substantial resource impact.

### 1.2 Pharmacological interventions

Two RCTs (online supplemental table 3) were included in the review^19 20^ to address the efficacy of proton pump inhibitors (PPIs) and aspirin for chemoprevention. Both the studies included people with low-grade dysplasia in Barrett’s oesophagus.

**Figure 1:**
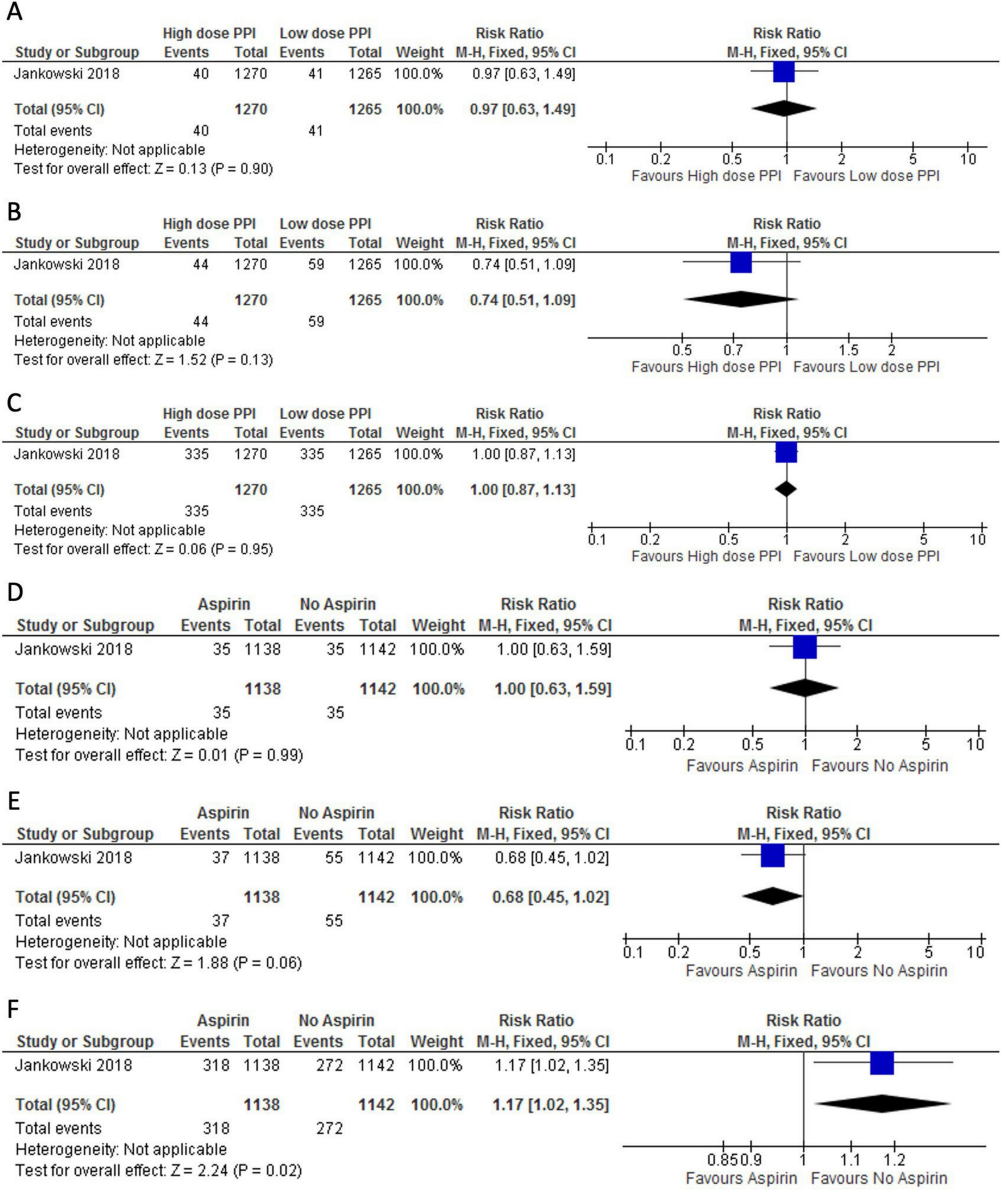
Evidence on pharmacological treatment for Barrett’s oesophagus. Forest plot of the association between pharmacological treatments with proton pump inhibitors (PPIs) (A–C) or aspirin (D–F) and development of oesophageal adenocarcinoma (A, D), high-grade dysplasia (B, E) or adverse events (C, F). Only one study could be included in the analysis.

This limited evidence, including the results from a large UK-based randomised trial (AspECT) showed that high-dose PPIs had no clinically important effect on outcomes (including all-cause mortality, progression to any grade of dysplasia or cancer, and serious adverse events) (figure 1A,B). The committee discussed that, although treatment with PPIs might potentially have chemopreventive effects against dysplasia and oesophageal adenocarcinoma compared with no treatment based on meta-analyses of retrospective studies,^21 22^ this would be difficult to demonstrate within a clinical trial setting because a placebo-controlled trial is not feasible as most people with Barrett’s oesophagus need treatment with PPIs for control of acid reflux symptoms. There was consensus that the current evidence did not support a recommendation for the use of PPIs to prevent progression to dysplasia and oesophageal cancer. The committee emphasised that a higher dose of PPIs was not associated with a higher number of adverse events or increased all-cause mortality (figure 1C). They noted that the current evidence does not justify a recommendation for high-dose PPIs, but agreed, based on clinical experience, that acid-suppressant medication such as PPIs should be offered to all patients to control symptoms of gastro-oesophageal reflux; however, the dose should be reviewed regularly to assess for side effects and prevent potential long-term side effects such as bone fractures, infections and electrolyte disturbances.^23^ Therefore, since the main goal for acid-suppressing treatment should be symptom control, the committee agreed to cross reference to the recommendations on managing gastro-oesophageal reflux disease in the NICE guideline on gastro-oesophageal reflux disease and dyspepsia in adults.

The committee agreed there was insufficient evidence from the AspECT trial to recommend aspirin to prevent progression to oesophageal dysplasia and cancer (figure 1D,E) and decided to make a ‘do not offer’ recommendation. Evidence from the AspECT trial showed that participants taking aspirin were more likely to have adverse events than those who were not, but the difference was small and not conclusive (figure 1F). The committee noted that this was in line with their clinical experience and knowledge that bleeding is more likely to be seen in people treated with aspirin. They agreed that the inconclusive results could be attributed to a protective effect from PPIs against bleeding both in the aspirin and no-aspirin study groups. Aspirin is not currently used to prevent progression to oesophageal dysplasia and cancer. Therefore, the recommendations are not expected to result in a change in current practice or to have an impact on resources.

### 1.3 Endoscopic surveillance

**Figure 2:**
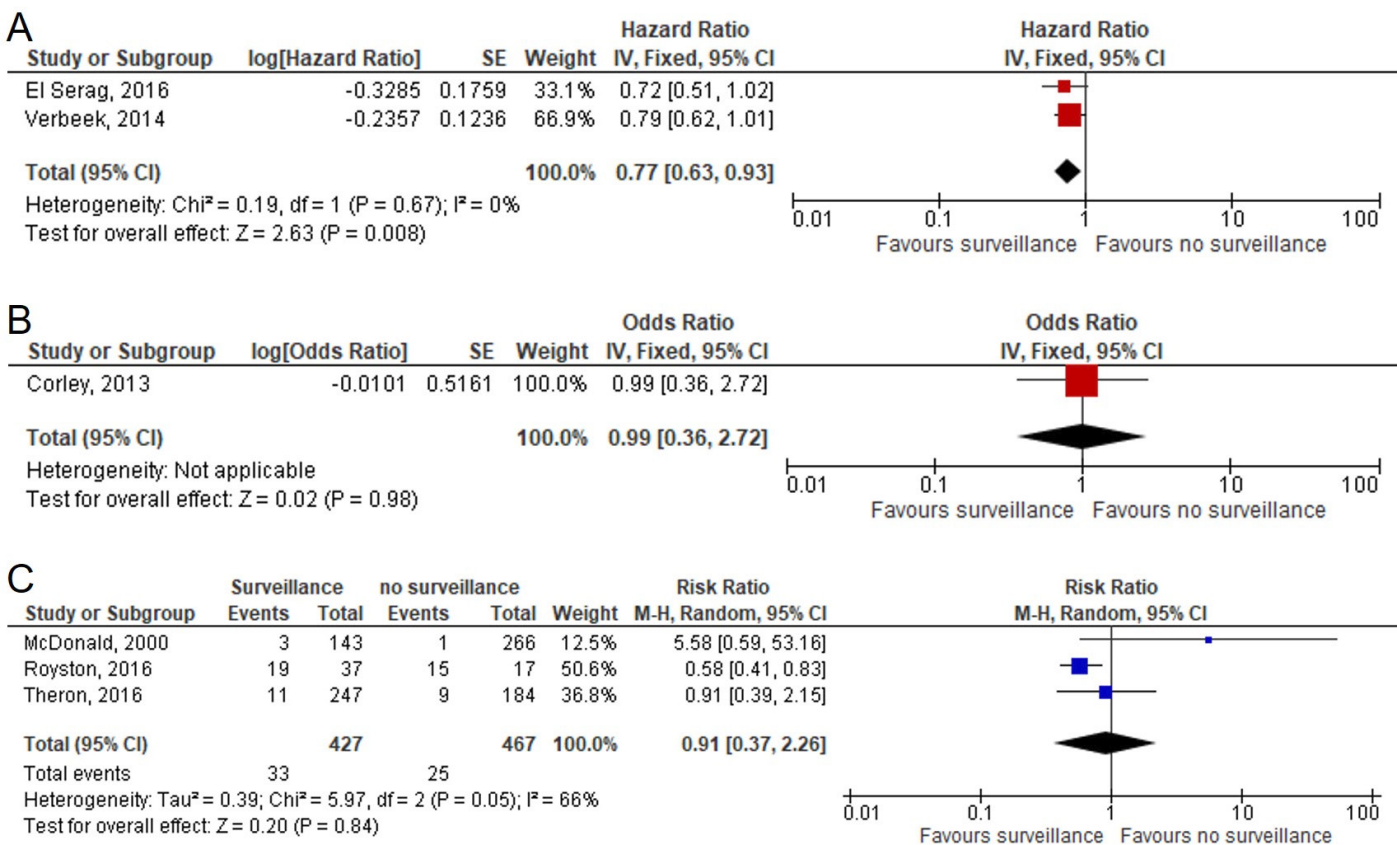
Effect of endoscopic surveillance on mortality by oesophageal adenocarcinoma. Forest plot of the association between endoscopic surveillance and mortality by oesophageal adenocarcinoma. (A) Adjusted studies with available data on the quality of surveillance. (B) Adjusted study with data on quality of endoscopy not available. (C) Unadjusted studies.

We assessed the effectiveness of endoscopic surveillance on patients' outcome, including mortality, neoplastic progression, stage of disease and quality of life. In the absence of randomised controlled data, the committee used the evidence from seven observational (online supplemental table 4) studies.^24–30^ The quality of the evidence was rated as low or very low. One reason for this was serious or critical risk of bias for the majority of the outcomes. Serious or critical risk of bias resulted from selection bias in studies due to their observational design. Although three studies used some form of statistical adjustment to reduce potential bias, this is unlikely to have reduced selection bias to the levels expected in randomised studies. The evidence from two adjusted observational studies showed that endoscopic surveillance using high-resolution white light reduced disease-specific and all-cause mortality, lowering the risk by almost 30% compared with no surveillance (figure 2A). However, one study showed a contradictory result, demonstrating no difference in the odds of mortality between surveillance and no surveillance (figure 2B). One explanation for this contradiction was an uncertainty about the quality of surveillance performed in the latter study, where the adequacy of endoscopic surveillance was not reported. In the other studies that reported mortality, no attempts were made to reduce confounding factors, leading to critical risk of bias (figure 2C)

There were also four economic evaluations reporting results for endoscopic surveillance versus no surveillance.^31–34^ One reported it being dominated by no surveillance, which the committee agreed was based on implausible health state utility scores and dated costs.^31^ One was based around surveillance according to the Seattle protocol and reported the cost per quality-adjusted life-year (QALY) as being greater than £20 000.^32^ The final two reported that it was cost effective at a cost per QALY threshold of £20 000. The first of these was based on a stratified population, whereas the second was based on a general population.^33 34^ The scheduling of surveillance in the latter study resembled UK guidelines.

Based on the clinical and cost-effectiveness evidence and considering the committee members' clinical experience, the committee agreed that endoscopic surveillance should be offered to people with Barrett’s oesophagus, provided that the person’s general health was adequate, and the benefits of surveillance outweighed the risks. The committee noted that this is the current standard of care for endoscopic surveillance for Barrett’s oesophagus.

The committee agreed that the risk of complications of endoscopic surveillance should be considered on an individual basis because the frequency and consequences of complications will vary depending on a range of factors, including age, frailty and medical comorbidities. It was agreed that possible complications should be discussed with the person with Barrett’s oesophagus.

In terms of diagnostic accuracy for Barrett’s related neoplasia, evidence for electronic and conventional chromoendoscopy techniques (including narrow-band imaging, acetic acid, methylene blue, autofluorescence imaging, confocal endomicroscopy, artificial intelligence) as well as endoscopic brushing (WATS3D) was obtained from people with dysplasia (online supplemental table 5) and early-stage cancer.^35–49^ This means that these techniques have not been validated in an unselected population with Barrett’s oesophagus undergoing standard endoscopic surveillance and therefore could not be recommended for routine application. A recommendation for research was made to assess the effectiveness of these techniques for surveillance of Barrett’s oesophagus.

### Frequency and duration of endoscopic surveillance

There was no evidence to support an optimal frequency for endoscopic surveillance as this will differ according to individual risk factors. However, the committee agreed to make a recommendation for frequency of surveillance based on length of segment, in line with the British Society of Gastroenterology guidelines and current practice in the UK (endoscopy every 2-3 years for Barrett’s oesophagus 3cm or longer and every 3-5 years for Barrett’s oesophagus <3 cm).^8^

The committee agreed that having a range for the surveillance interval allows tailoring the frequency of surveillance to each person based on a clinical assessment of their risk of cancer, with length of segment, being the factor most closely linked to risk of cancer, but age, sex, family history of oesophageal cancer and smoking also being important.^9 50 51^ In clinical practice shorter intervals can be considered for patients perceived at higher risk—for example, those affected by very long segments of Barrett’s oesophagus, those who are older, male and with a positive family history. Whereas longer intervals can be recommended for younger individuals and those with no additional risk factors of progression.

In line with current practice, there was consensus that people with short-segment (<3 cm) Barrett’s oesophagus without intestinal metaplasia (confirmed at two endoscopies) should not be offered endoscopic surveillance because the risk of disease progression is low in this population and there are risks associated with endoscopic surveillance.^9 52^

There was no evidence on the duration of endoscopic surveillance, and the committee agreed not to make a recommendation for this.

Endoscopic surveillance is widely used for monitoring people with Barrett’s oesophagus. Adherence to the biopsy protocols requires additional procedure time beyond that of a standard endoscopy, but many services have already increased the time allocation for Barrett’s surveillance and the overall impact on resources is not expected to be significant.

In the absence of clinical evidence for people with indefinite dysplasia of the oesophagus, the committee drew on their clinical experience to make a recommendation for this population. They emphasised that the risk of progression to high-grade oesophageal dysplasia or cancer is around three to five times higher than the risk in the non-dysplastic population,^53 54^ and therefore endoscopic surveillance at 6 months would be appropriate. The committee also noted, based on their clinical experience, that indefinite dysplasia is often linked to excessive inflammation of the oesophagus and, therefore dose escalation of acid-suppressant medication is appropriate. The committee agreed that if no definite dysplasia is confirmed at follow-up endoscopy, the surveillance interval should return to the appropriate one for non-dysplastic Barrett’s oesophagus.

People with low-grade dysplasia should be closely monitored at a 6- month time interval. See section 1.5 for discussion on the management of people with low-grade dysplasia.

The committee emphasised that evidence of clinical and molecular biomarkers associated with a greater risk of progression to dysplasia or cancer could inform setting appropriate intervals for endoscopic surveillance and agreed to make a recommendation for research on biomarkers.

### Non-endoscopic surveillance techniques (no recommendations)

There was evidence of benefit of using cytosponge to diagnose dysplasia and cancer, but the quality was not sufficient to support its use at present. Cytosponge with laboratory biomarkers had high diagnostic accuracy in detecting high-grade dysplasia/cancer and any grade of dysplasia/cancer, but the sensitivity was not high enough to meet the clinical decision threshold.^55^ The committee noted that cytosponge is more likely to be used in a lower-risk group. While agreeing that the results for diagnostic accuracy looked promising, these results were based on a single non-randomised retrospective study and therefore conclusions could not be drawn with certainty. The committee acknowledged that the use of cytosponge might be an option for patients not wanting endoscopy. Patients who have had cytosponge often prefer it^56^ and it has the potential to reduce the pressure in endoscopy services if found to be effective. However, the committee emphasised that the current evidence does not support its implementation in current practice and agreed it could not be recommended at the current time. The committee also agreed it would not be appropriate to make a research recommendation because of ongoing trials, and the evidence could be reviewed once they have been completed.

Balloon brushing is an old technique that is not currently used in clinical practice.^57^ Limited evidence on cytology obtained from balloon brushing showed it could detect oesophageal dysplasia and adenocarcinoma, but the committee agreed there was insufficient evidence to recommend its use in clinical practice.

There was a lack of evidence on other non-endoscopic surveillance techniques, such as Esophacap and Esoguard, and based on their clinical experience, the committee agreed it was not appropriate to recommend them.

### 1.4 Staging for suspected stage 1 oesophageal adenocarcinoma

Five studies (online supplemental table 6) were included which covered different imaging techniques (mini-probe EUS and radial EUS and CT).^58–62^

The committee agreed, based on their clinical experience, that the diagnostic accuracy of CT is very low for detecting stage 1 oesophageal adenocarcinoma because of the resolution of the technique, due to the small size of T1 oesophageal adenocarcinoma. Evidence showed that CT had a high specificity (1.00) but a very low sensitivity (0.38) for N staging. Therefore, there was consensus that CT should not be used before endoscopic resection for staging suspected T1 oesophageal adenocarcinoma.

Evidence for the accuracy of mini-probe EUS in detecting T1a versus T1b tumours showed a high sensitivity (0.89) almost reaching the clinical threshold of 0.9, but a low specificity (0.27) that did not reach the threshold of 0.8 (online supplemental table 7). The committee noted that mini-probe EUS performed well in detecting T1a but not T1b tumours. The diagnostic accuracy of the mini-probe EUS for N staging was higher with a sensitivity of 0.75 and a specificity 0.97 that exceeded the clinical threshold. The diagnostic accuracy of conventional radial endoscopic ultrasonography (crEUS) for distinguishing T1a (vs T1b) tumours and T1b (vs T1a) in people with early cancer was moderately high, with measures of both sensitivity and specificity ranging between 0.64 and 0.78, but not high enough to reach the agreed thresholds for decisionmaking. However, the diagnostic accuracy for detecting N1 status was very high with sensitivity (1.00) and specificity (0.92) both exceeding clinical thresholds set for decision-making. For distinguishing T1b from T0 and T1a, in people with high-grade dysplasia or cancer, crEUS had a sensitivity of 0.56, suggesting that EUS is not reliable in differentiating T1a with T1b tumours. The committee confirmed based on their clinical experience that crEUS can be useful in people with suspected T1b based on endoscopic appearances to exclude T2 stage or higher, but noted that, since the current result was based on only five cases with T1b, there was imprecision in the effect estimate and the population was partially indirect due to the inclusion of a small number of people with T2 and T3 in the analysis. The committee agreed that EUS should not be used before endoscopic resection for staging suspected T1a oesophageal adenocarcinoma, as this carries a negligible risk of lymph node metastasis. EUS should be considered when an oesophageal lesion is suspected to be T1b cancer based on endoscopic appearances—for example, sessile lesions with significant luminal component (Paris 0–Is) or depressed lesions (Paris 0–IIc). It should also be considered for people with confirmed T1b oesophageal adenocarcinoma based on endoscopic resection staging, who have a significant risk of lymph node metastasis and might benefit from additional surgery or oncological treatment, such as radiotherapy alone or in combination with chemotherapy.

Based on their clinical experience, the committee also noted that PET, CT and EUS can all over-stage tumours, but EUS has the advantage of providing pathological confirmation for the presence of involved lymph nodes.

In the absence of definitive evidence on endoscopic staging techniques, the committee drew on their clinical experience to inform decision-making. They agreed that endoscopic resection should be offered to people with suspected stage 1 oesophageal adenocarcinoma as it is the most accurate staging technique and is the gold standard in current practice as recommended by the British Society of Gastroenterology guidelines.

These recommendations are in line with current practice and therefore will not have an impact on resources.

### 1.5 Managing Barrett’s oesophagus with low-grade dysplasia

**Figure 3:**
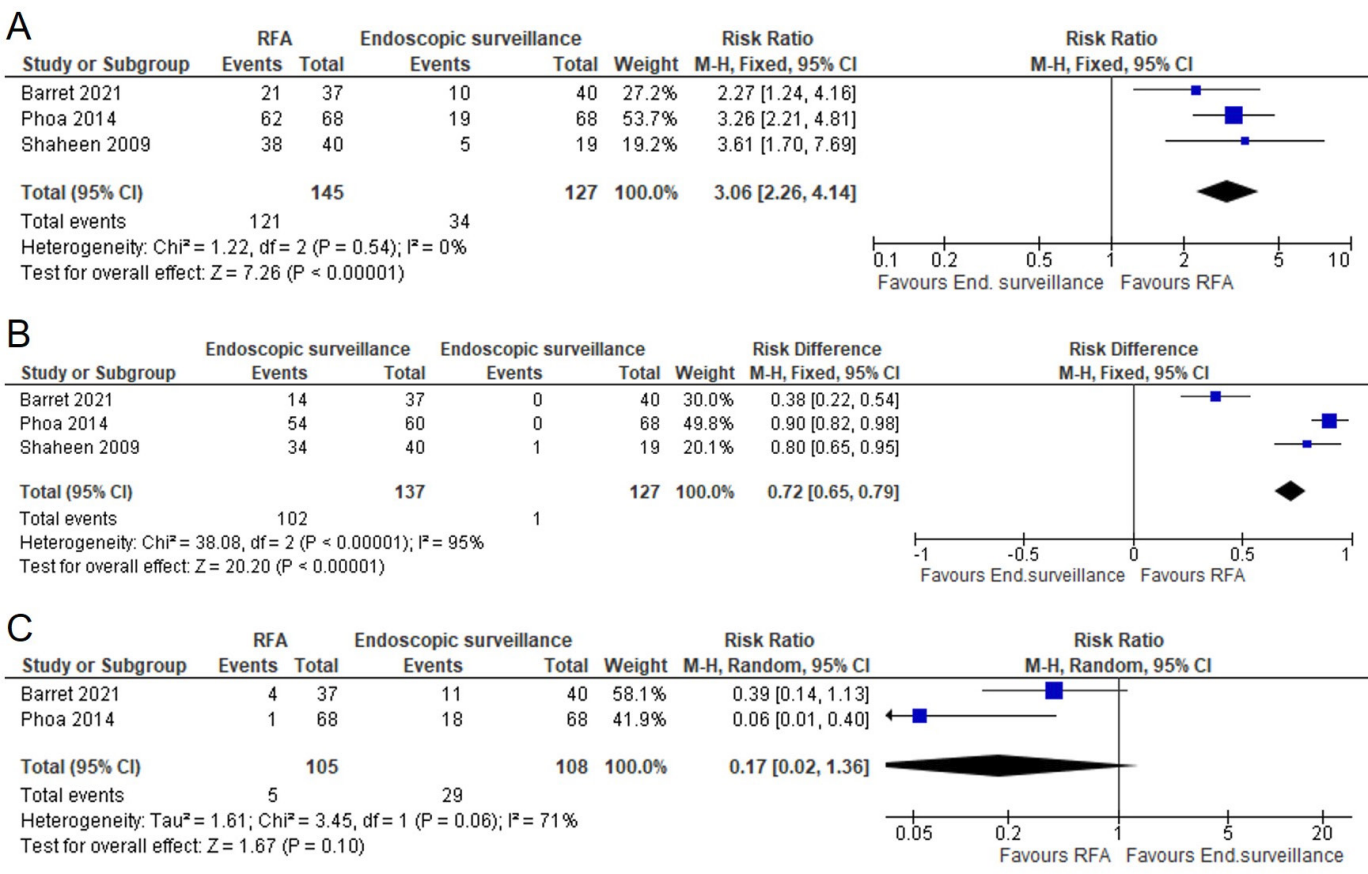
Effectiveness of radiofrequency ablation (RFA) in people with low-grade dysplasia. Forest plot assessing the effect of RFA in people with low-grade dysplasia on resolution of dysplasia (A), resolution of intestinal metaplasia (B) and progression to high-grade dysplasia or cancer (C).

Clinical evidence from three RCTs (online supplemental table 8) and one observational study^63–66^ showed a clinically important benefit of radiofrequency ablation (RFA) compared with endoscopic surveillance across all outcomes examined in patients with Barrett’s oesophagus and low-grade dysplasia, except for complications. RCT evidence showed a clinically important benefit of RFA over endoscopic surveillance for complete eradication of dysplasia and complete eradication of intestinal metaplasia (figure 3A,B).

Evidence from these studies showed that in people with confirmed low-grade oesophageal dysplasia, RFA protects from progression to high-grade dysplasia or cancer (figure 3C).

A cost-effectiveness analysis comparing RFA with endoscopic surveillance reported the cost per progression to neoplasia prevented.^67^ The committee noted that it was difficult to decide on an acceptable threshold at which the cost per progression averted would represent value for money. A cost–utility analysis also compared RFA with endoscopic surveillance over 15 years and showed that RFA was cost effective at a threshold of £20 000 per QALY gained.^68^

Based on their clinical experience and inclusion criteria for available RCTs, the committee emphasised that for RFA to be offered, evidence of low-grade dysplasia from biopsy results from two separate endoscopies and confirmation of the diagnosis on both sets of biopsies by two gastrointestinal pathologists should be present. They noted this was in line with current practice where RFA takes place in specialist centres by endoscopists with appropriate experience and would not be considered in cases where there is evidence of low-grade oesophageal dysplasia from biopsy results from only one endoscopy or where there is no confirmation by a second gastrointestinal pathologist.

There was no evidence to support use of other ablation techniques for treating low-grade dysplasia.

### 1.6 Managing high-grade dysplasia and stage 1 oesophageal adenocarcinoma

Eight studies (online supplemental table 9) (six RCTs, two observational studies) were included in the systematic review.^66 69–75^ The studies compared different endoscopic treatments. RCT evidence was identified on the following topics: Argon plasma coagulation (APC) compared with surveillance; endoscopic resection (ER) combined with APC compared with ER combined with RFA; ER using a cap with snare compared with multi-band mucosectomy; RFA compared with sham endoscopic procedure; endoscopic submucosal dissection (ESD) compared with endoscopic mucosal resection (EMR); focal ER combined with stepwise radical ER compared with focal ER combined with RFA.

Observational evidence was identified comparing EMR combined with RFA with RFA alone and RFA with cryotherapy.

The evidence showed that endoscopic treatment using a combination of endoscopic resection and endoscopic ablation for visible dysplastic lesions or endoscopic ablation alone for Barrett’s oesophagus without a visible lesion is effective to treat people with high-grade dysplasia and prevent progression to adenocarcinoma.

In addition to the clinical studies, four cost–utility analyses were identified.^68 76–78^ They reported that at a threshold of £20 000 per QALY gained, EMR combined with RFA was cost effective versus endoscopic surveillance^77^; EMR plus RFA plus surveillance, EMR plus APC plus surveillance and surgery were all cost effective versus no surveillance^78^; RFA followed by endoscopic surveillance was cheaper and more effective than oesophagectomy^76^; and RFA alone was cheaper and more effective than oesophagectomy.^68^

Based on clinical experience the committee recommended that high-grade dysplasia should be endoscopically resected, when oesophageal lesions are visible at endoscopy, and the residual Barrett’s oesophagus should be treated with endoscopic ablation.

There are two techniques for resection of dysplastic lesions in Barrett’s oesophagus: EMR and ESD. There is no evidence of superiority of one technique over the other so the recommendation does not specify which to use.^75^

The evidence indicated that both RFA and APC are effective in reducing the risk of recurrent dysplasia in people who have received an endoscopic resection for high-grade dysplasia. However, the committee noted that for a very long segment, Barrett’s oesophagus RFA might be more practical than APC, which has a significantly smaller ablation area per treatment application than RFA. Given that there is no evidence of superiority of one ablation technique over the other, the committee agreed further research was needed to determine the most effective endoscopic ablation technique to use and made a recommendation for research.

For treating stage 1 adenocarcinoma, six observational studies (online supplemental table 10) were found comparing the outcome of individuals treated with oesophagectomy or endoscopic resection.^79–84^ Overall, the evidence was difficult to evaluate given the lack of adjustment for selection bias in five out of the six observational studies. The single adjusted study by Prasad adjusted only for the outcomes of all-cause mortality and mortality related to oesophageal adenocarcinoma. This study showed no clear effect for all-cause mortality, but did demonstrate a very clear benefit for oesophagectomy in terms of mortality related to oesophageal adenocarcinoma. However, the committee noted the study’s limitations because of the age of that study, and that an older technique had been used for many of the patients in the endoscopic therapy arm. The study had been carried out in 2007, and the committee agreed that because this was before the formalisation of quality standards in endoscopic treatment in 2010 this might have underestimated endoscopic treatment benefits. Therefore, the adjusted evidence was not considered as reliable as it might otherwise have been by the committee.

**Figure 4:**
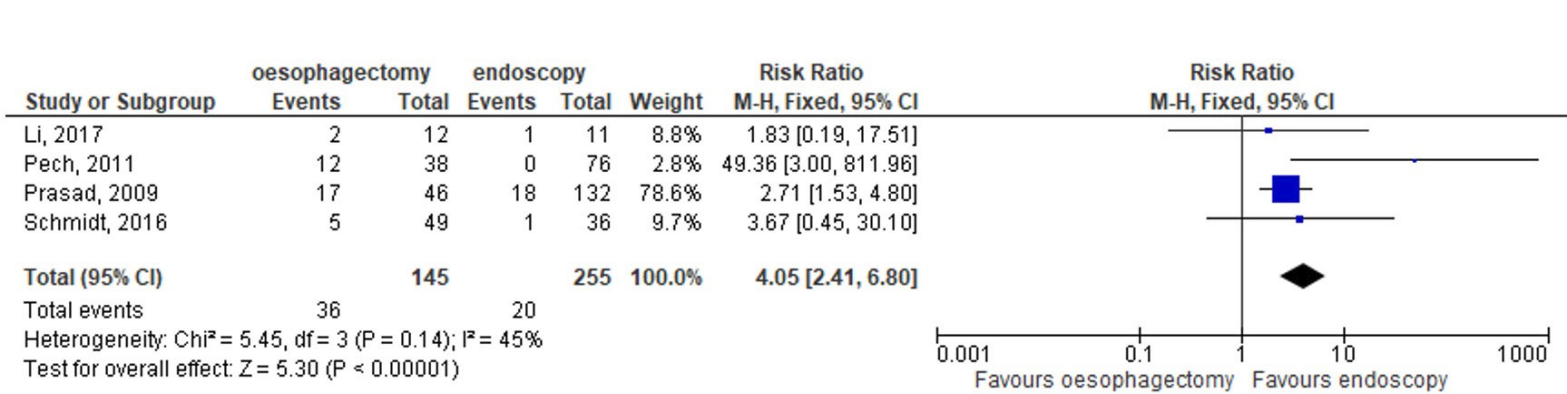
Major or serious complications of treatments for early oesophageal adenocarcinoma. Forest plot of the correlation of endoscopic or surgical treatment for high-grade dysplasia or oesophageal adenocarcinoma stage 1 and the occurrence of serious adverse events.

Overall, the quality of the evidence was limited but reflected the committee’s clinical experience that endoscopic resection and oesophagectomy are equally effective, but oesophagectomy is associated with a higher incidence of serious adverse events (figure 4). There was a lack of evidence on how the two treatments affect quality of life, so in considering this, the committee drew on their own experience. As part of standard practice, a clinical consultation should be offered to the patient to discuss the treatment options and the advantages and disadvantages of both approaches. Endoscopic resection is less invasive and has fewer complications than oesophagectomy.

The committee agreed that even after successful endoscopic treatment there remains a risk of recurrence of Barrett’s oesophagus and metachronous oesophageal neoplasia.^85 86^ Therefore, endoscopic treatment comes with a greater need for ongoing endoscopic surveillance, which could lead to anxiety about recurrence and possibly, impacts on the quality of life. This was reinforced by a lay committee member. Despite this, the committee agreed that endoscopic resection is still more likely to result in better quality of life post-treatment than oesophagectomy. Therefore, it should be offered as first-line treatment to people with T1a adenocarcinoma. The committee noted, based on their clinical experience, that ESD might offer an advantage in certain individuals with Barrett’s oesophagus-related neoplasia (lesions larger than 15 mm, poorly lifting tumours and lesions at risk for submucosal invasion) but there was no reason to select it routinely over EMR for small slightly elevated lesions because ESD is a complex procedure and is associated with more complications.

There was evidence supporting the effectiveness of using endoscopic ablation following endoscopic resection to treat people with T1a adenocarcinoma of the oesophagus arising in Barrett’s oesophagus. Similarly, to high-grade dysplasia, the evidence indicated that both RFA and APC are effective in reducing the risk of recurrent oesophageal neoplasia in people who have received an endoscopic resection for T1a adenocarcinoma. Given that there is no evidence of the superiority of one technique over the other, the committee agreed further research was needed to determine the most effective endoscopic ablation technique to use and made a recommendation for research.

Debate remains about the safest and most effective treatment for T1b oesophageal adenocarcinoma. Small cohort studies in patients with low-risk T1b adenocarcinoma (sm1 invasion, well-differentiated to moderately differentiated cancers and with no lymphovascular invasion) treated with endoscopic resection showed a rate of metastasis of approximately 2%.^87 88^ However, a Dutch nationwide study that analysed data from people treated surgically and endoscopically showed that the metastatic rate in low-risk T1b can be as high as 16% for cancers larger than 20 mm.^89^ A propensity-scored retrospective study based on a US National Cancer Database showed survival advantage for people with T1b adenocarcinoma treated with oesophagectomy compared with those treated with endoscopic resection only.^90^ Overall, the uncertainty about optimal treatment for T1b oesophageal adenocarcinoma was a concern for the committee. In the absence of prospective and well-conducted studies, the committee decided to make a recommendation to offer oesophagectomy rather than just endoscopic resection for people with T1b oesophageal adenocarcinoma at high risk of cancer progression. This was based on their clinical experience that there is a greater risk of local recurrence in cases of incomplete endoscopic resection and a high risk of lymph node metastasis in cases with deep submucosal invasion (>500 μm) and lymphovascular invasion.^89^ They decided not to make a recommendation for people with T1b at low risk of cancer progression as it was less clear which treatment option would be best, but made a recommendation for research to determine the effectiveness of endoscopic resection with or without adjuvant chemoradiotherapy and oesophagectomy for adults with T1b oesophageal adenocarcinoma based on pathological staging by endoscopic resection.

### Follow-up after endoscopic treatment

In the absence of evidence comparing endoscopic and radiological follow-up as well as the optimal frequency of monitoring, the committee drew on their clinical experience to make a recommendation. They agreed that endoscopic follow-up is needed for people who have received endoscopic treatment for dysplastic Barrett’s oesophagus and stage 1 oesophageal adenocarcinoma as the likelihood of recurrence is high. The committee noted this was in line with current practice.

Based on their clinical experience, the committee agreed that the frequency of follow-up should be based on the likelihood of recurrence. In the absence of evidence, the committee decided to make a recommendation for research to assess the optimal frequency and duration of endoscopic follow-up for people who have received endoscopic treatment for stage 1 oesophageal adenocarcinoma.

The current recommendations are in line with current practice and therefore will not have a resource impact.

### 1.7 Non-surgical treatment for T1b oesophageal adenocarcinoma

No evidence could be identified. In the absence of evidence to guide decision-making, the committee drew on their clinical experience to make a recommendation on non-surgical treatment for T1b oesophageal adenocarcinoma.

Using radiotherapy alone or in combination with chemotherapy to treat oesophageal adenocarcinoma is current practice.^91^

The committee agreed that radiotherapy alone or in combination with chemotherapy would be appropriate for people with T1b oesophageal adenocarcinoma at high risk of cancer progression, based on staging endoscopic resection, as it is likely to reduce the risk of recurrence. The decision for further non-surgical treatment should be based on patient factors, including risk features on pathological staging, fitness and patient preference. They noted that chemotherapy alone is not a definitive treatment.

### 1.8 Anti-reflux surgery

Two small RCTs (online supplemental table 11) comparing anti-reflux surgery (Nissen fundoplication) with esomeprazole were included^92 93^ ; one RCT addressing the protocol outcome of adverse events, and one RCT on progression to high-grade dysplasia, dysplasia de novo (progression to any grade of dysplasia from non-dysplastic Barrett’s oesophagus) and complications such as splenectomy, inability to belch or vomit and transient postoperative dysphagia.

The evidence showed a clinically important benefit of medical treatment for the outcome of complications and no clinically important difference for the outcomes of progression to high-grade dysplasia and treatment failure. The committee agreed that the findings were in line with their clinical experience that anti-reflux surgery does not offer any advantage over medical treatment with PPIs for progression to dysplasia or cancer. Therefore, the committee agreed, based on the current limited and low quality of evidence that anti-reflux surgery cannot be recommended for chemoprevention for people with Barrett’s oesophagus.

The committee discussed that although PPIs are widely used in current practice for symptom control in people with Barrett’s oesophagus, there are a number of people who express concerns about being on high-dose PPI medication long term, or who are intolerant to the medication. The committee agreed that anti-reflux surgery provides an alternative option to long-term medical treatment for this group of people.

One observational study was identified comparing anti-reflux surgery with medical treatment (esomeprazole) after RFA. The quality of the evidence was very low; therefore the committee had low confidence in the quality of the evidence as it came from an observational study with very wide confidence intervals and a very small number of participants. People who do not respond to RFA are sometimes referred for anti-reflux surgery. However, the committee noted that in such cases other ablation therapies, such as APC, could be considered instead of anti-reflux surgery.

### 1.9 Future research

We recognised that there are important topics for future research. In making research recommendations we considered known ongoing trials to avoid duplication of efforts and prioritised topics that are more likely to affect clinical management and have resource implications. We took into account areas where there is a historical lack of evidence in support of clinical guidelines. We have identified the following research questions that we hope the research community will address in the future to help manage patients with Barrett’s oesophagus

- What is the diagnostic accuracy for early neoplasia of different endoscopic surveillance techniques, including high-resolution endoscopy and chromoendoscopy, in adults with Barrett’s oesophagus?

- What is the usefulness of clinical and molecular biomarkers to inform the optimal frequency and duration of endoscopic surveillance for adults with Barrett’s oesophagus?

- What is the effectiveness of endoscopic resection with or without adjuvant chemoradiotherapy and oesophagectomy for adults with T1b oesophageal adenocarcinoma?

- For adults with Barrett’s oesophagus with dysplasia and stage 1 oesophageal adenocarcinoma, what is the effectiveness of different endoscopic ablation techniques alone or in combination with endoscopic resection?

- What is the optimal frequency and duration of endoscopic follow-up for patients who have received endoscopic treatment for Barrett’s oesophagus with dysplasia and stage 1 oesophageal adenocarcinoma?
