# Supplementary material for: National Institute for Health and Care Excellence (NICE) guidance on monitoring and management of Barrett’s oesophagus and stage I oesophageal adenocarcinoma
Source: Gut. 2024 Mar 29;73(6):897–909. doi: 10.1136/gutjnl-2023-331557 (PMC11103346; doi:10.1136/gutjnl-2023-331557)
Supplement: Supplementary data [file gutjnl-2023-331557supp001.pdf]

Supplementary Tables, di Pietro et al, National Institute for Health and Care Excellence (NICE) guidance on monitoring and management of Barrett's oesophagus and stage I oesophageal adenocarcinoma

Supplementary Table 1: list of review questions

Type of review	Review questions	Outcomes
Qualitative	What information and support should be provided to patients (or carers or families) who are having or considering follow-up or treatment for Barrett's oesophagus or stage 1 adenocarcinoma?	• Themes emerging from qualitative data (themes were derived from the evidence identified for this review and not pre-specified)
Intervention	For adults with Barrett's oesophagus, what is the clinical and cost effectiveness of pharmacological interventions (such as antacids, aspirin, H2 receptor antagonists, proton pump inhibitors) in reducing progression to dysplasia or cancer?	• Mortality (including all-cause mortality)<br>• Health related quality of life<br>• Progression from non-dysplastic to low grade dysplasia<br>• Progression to any grade of dysplasia<br>• Progression to high grade dysplasia or cancer<br>• Adverse events (e.g., bleeding)
Intervention	For adults with Barrett's oesophagus, what is the clinical and cost effectiveness of endoscopic surveillance using white light endoscopy?	• Mortality<br>• Health related quality of life (validated scores)<br>• Progression of dysplasia<br>• Progression to cancer and stage<br>• Adverse events (such as sedation related, bleeding, pain, perforation)
Diagnostic test accuracy	What is the diagnostic accuracy of different endoscopic surveillance techniques including high resolution endoscopy and chromoendoscopy?	• Detection of progression of dysplasia<br>• Sensitivity<br>• Specificity

Supplementary Tables, di Pietro et al, National Institute for Health and Care Excellence (NICE) guidance on monitoring and management of Barrett's oesophagus and stage I oesophageal adenocarcinoma

Type of review	Review questions	Outcomes
		• Data to calculate 2x2 tables to calculate sensitivity and specificity (number of true positives, true negatives, false positives and false negatives).
Intervention<br><br>Diagnostic test accuracy	For adults with Barrett's oesophagus, what is the clinical and cost effectiveness of different non-endoscopic surveillance techniques, including cytosponge?<br><br>What is the diagnostic accuracy of different non-endoscopic surveillance techniques including cytosponge?	• Detection of any grade of dysplasia<br>• Detection of early cancer or high-grade dysplasia<br>• Health related quality of life<br>• Adverse events (bleeding, perforation, pain)<br>• Rate of inadequate sampling (requiring repeat or conversion<br>• Detection of progression to any grade of dysplasia<br><br>• Sensitivity<br>• Specificity<br>• Data to calculate 2x2 tables to calculate sensitivity and specificity (number of true positives, true negatives, false positives and false negatives).
Intervention	What is the optimal frequency and duration of endoscopic surveillance for adults with Barrett's oesophagus?	• Health related quality of life<br>• Progression to high grade dysplasia or cancer<br>• Mortality<br>• Adverse events / complications (bleeding, perforation, pain)<br>• Adherence to surveillance (physician and patient)
Diagnostic test accuracy	Diagnostic test accuracy<br><br>For adults with suspected stage 1 carcinoma, what is	• Diagnostic test accuracy<br>• Tumour or Node or Metastasis staging (or all)<br>• Sensitivity<br>• Specificity

Supplementary Tables, di Pietro et al, National Institute for Health and Care Excellence (NICE) guidance on monitoring and management of Barrett's oesophagus and stage I oesophageal adenocarcinoma

Type of review	Review questions	Outcomes
	the clinical and cost effectiveness of different endoscopic and radiological staging techniques?	• Data to calculate 2x2 tables to calculate sensitivity and specificity (number of true positives, true negatives, false positives and false negatives)
Diagnostic RCT	For adults with suspected stage 1 adenocarcinoma, what is the diagnostic accuracy of different endoscopic and radiological staging techniques?	• Health-related quality of life<br>• Progression to higher stage of cancer<br>• Mortality<br>• Adverse events (staging perforation, bleeding, pain, allergic reaction to contrast and complications of oesophagectomy)
Intervention	For adults with Barrett's Oesophagus with low grade or indefinite dysplasia, what is the clinical and cost effectiveness of endoscopic treatments?	• Mortality (disease specific mortality and all-cause mortality)<br>• Health related quality of life<br>• Complete regression of Barrett's dysplasia and Barrett's oesophagus<br>• Recurrence of dysplasia or neoplasia<br>• Need for retreatment<br>• Complications of treatment (bleeding, perforation, stricture, pain)<br>• Rate of hospitalization<br>• Progression to higher grade dysplasia and cancer<br>• Conversion to non-endoscopic procedure
Intervention	For adults with high-grade dysplasia and stage 1 adenocarcinoma, what is the clinical and cost	• Mortality (disease specific mortality and all-cause mortality)<br>• Treatment related mortality<br>• Health related quality of life (any validated score)<br>• Complete regressions of dysplasia or Barrett's oesophagus<br>• Recurrence of Barrett's dysplasia or neoplasia

Supplementary Tables, di Pietro et al, National Institute for Health and Care Excellence (NICE) guidance on monitoring and management of Barrett's oesophagus and stage I oesophageal adenocarcinoma

Type of review	Review questions	Outcomes
	effectiveness of endoscopic treatments alone or in combination?	• Need for retreatment<br>• Complications of treatment (e.g. bleeding, pain infection, perforation, stricture)<br>• Length of hospital stay<br>• Conversion of endoscopic treatment to surgery
Intervention	For adults with stage 1 oesophageal adenocarcinoma, what is the clinical and cost effectiveness of oesophagectomy?	• Mortality (all-cause mortality, disease specific and treatment related)<br>• Health related quality of life (any validated scores)<br>• Progression of stage 1 adenocarcinoma to higher stages<br>• Complications of surgery (e.g. perforation, stricture, pneumonia, anastomotic leak, weight loss, sepsis)<br>• Adverse events (e.g. stricture, chronic ill health, chronic pain)<br>• Length of hospital stay<br>• Regression of Barrett's Oesophagus<br>• Recurrence of Barrett's Oesophagus and Barrett's related neoplasia<br>• Repeat intervention<br>• (need for) Conversion from endoscopic to surgery
Intervention	For adults with stage 1b adenocarcinoma, what is the clinical and cost effectiveness of different non-surgical interventions?	• Mortality (all-cause mortality & disease specific mortality)<br>• Health related quality of life (any validated score)<br>• Progression of stage 1 adenocarcinoma to higher stages<br>• Severe adverse events from oncological treatment. Such as: Infection, Thrombosis, Myelosuppression, Cardiac or respiratory complications, Radiation stricture or fistula, GI disease effects (diarrhoea, nausea, vomiting)<br>• Adverse events from surgery & endoscopic treatment

Supplementary Tables, di Pietro et al, National Institute for Health and Care Excellence (NICE) guidance on monitoring and management of Barrett's oesophagus and stage I oesophageal adenocarcinoma

Type of review	Review questions	Outcomes
Intervention	For adults with Barrett's Oesophagus, what is the clinical and cost effectiveness of anti-reflux surgery to reduce progression to dysplasia or cancer?	• Mortality (disease specific mortality, treatment related mortality and all cause)<br>• Health related quality of life<br>• Dysphagia<br>• Progression to/of dysplasia<br>• Progression to cancer<br>• Adverse events (including failure of procedure, rate of re-operation, sedation related, bleeding, pain, perforation)<br>• Reintroduction of regular medication<br>• Rate of re-introduction of PPI
Intervention	For adults with Barrett's Oesophagus or stage 1 adenocarcinoma, what is the clinical and cost effectiveness of anti-reflux surgery to induce remission of disease or prevent recurrence?	• Mortality (disease-specific mortality, treatment related mortality and all cause)<br>• Health related quality of life<br>• Progression of grade of dysplasia<br>• Progression to cancer<br>• Recurrence of Barrett's oesophagus/ dysplasia/cancer<br>• Number of endoscopic treatments to achieve remission of Barrett's<br>• Time duration of the endoscopic treatment<br>• Adverse events (such as bleeding, pain)
Intervention	For people who have received endoscopic treatment for Barrett's Oesophagus related stage 1 adenocarcinoma, what is the clinical and cost	• Mortality (all-cause mortality and disease specific mortality)<br>• Health related quality of life (any validated scores)<br>• Recurrence of cancer or dysplasia<br>• Adverse events (infection, perforation, bleeding)<br>• Detection of incidental findings and subsequent investigations

Supplementary Tables, di Pietro et al, National Institute for Health and Care Excellence (NICE) guidance on monitoring and management of Barrett's oesophagus and stage I oesophageal adenocarcinoma

Type of review	Review questions	Outcomes
	effectiveness of endoscopic follow up with or without radiological follow up?	
Intervention	For people who have received endoscopic treatment for Barrett's oesophagus or stage 1 adenocarcinoma, what is the optimal frequency and duration of endoscopic and radiological follow up?	• Mortality (all cause and disease specific mortality)<br>• Health related quality of life (any validated scores)<br>• Patient preference<br>• Recurrence of Barrett's Oesophagus<br>• Recurrence Stage 1 adenocarcinoma<br>• Adverse events (stricture, perforation, infection, bleeding)<br>• Endoscopic reintervention<br>• Non endoscopic intervention (oncological or surgical)

Supplementary Tables, di Pietro et al, National Institute for Health and Care Excellence (NICE) guidance on monitoring and management of Barrett's oesophagus and stage I oesophageal adenocarcinoma

Supplementary Table 2: Summary of studies included in the evidence review for patient information and support

Study	Design	Population	Research aim	Comments
Arney 2014	Structured, in-depth qualitative interviews with framework analysis methodology.	Barrett's oesophagus patients (n=20) who had received at least one surveillance esophagogastroduodenoscopy (EGD).<br><br>Mean age (SD): 62.9 (7.32) years; n=9 were diagnosed with no dysplasia, n=10 with low-grade dysplasia and n=1 with high-grade dysplasia.<br><br>USA	To define the patient experience of EGD from in-depth qualitative interviews with patients who recently underwent surveillance EGD.	35% completed five or more surveillance EGDs; 25% completed only 1 prior EGD.<br><br>Mean number (range) of completed EGDs: 4.3 (1-20).<br><br>None of the EGD procedures were performed using propofol or monitored anaesthesia care; 90% were performed under conscious sedation combined with topical anaesthesia to the back of the throat.
Bailey 2009	Semi-structured interviews with thematic analysis	Patients enrolled on a Barrett's surveillance programme in a large teaching hospital. (N=15)<br><br>Median age: 59 years (36-77)	To assess patients' perceptions, experience, and informational needs about Barrett's oesophagus to improve the understanding of health professionals and	Participants had been on a surveillance programme for a combined total of 92 years, with a mean of 6 years (ranging from 1–21 years).<br><br>Eleven participants (73%) were receiving

Supplementary Tables, di Pietro et al, National Institute for Health and Care Excellence (NICE) guidance on monitoring and management of Barrett's oesophagus and stage I oesophageal adenocarcinoma

Study	Design	Population	Research aim	Comments
		UK	therefore their ability to provide the best care for their patients.	2-yearly surveillance endoscopies.
Britton 2019	Exploratory qualitative research, part of a concurrent mixed-methods study involving semi-structured interviews with thematic analysis	Patients with Barrett's oesophagus, enrolled in surveillance at a single general NHS hospital (N=20)<br><br>Median age= 63 years (42-77 y)<br><br>UK	To identify and explore factors impacting BO patients' HRQOL, the follow-up needs of BO patients and patients' perceptions and attitudes to new models of follow-up care	Participant's demographics and disease-specific information were also collected from their medical notes and endoscopy reports.<br>Field notes were taken at the time of each interview.
Cooper 2009	Questionnaire study with quantitative analysis	Barrett's oesophagus patients undergoing surveillance (n=151)<br><br>Median age (range): 66 (41-79).	To examine the experience of patients undergoing endoscopic surveillance for BO, their levels of anxiety and depression, and	The questionnaire included seven questions on patients' attitudes towards Barrett's oesophagus and endoscopic surveillance, and perceptions of cancer

Supplementary Tables, di Pietro et al, National Institute for Health and Care Excellence (NICE) guidance on monitoring and management of Barrett's oesophagus and stage I oesophageal adenocarcinoma

Study	Design	Population	Research aim	Comments
		No dysplasia 90%, indefinite dysplasia 3%, low-grade dysplasia 7%, high-grade dysplasia 0%<br><br>UK	quality of life and how the relationship with their physicians influences these factors.	risk in Barrett's oesophagus.
Gough 2003	Postal questionnaire with quantitative analysis	Barrett's oesophagus patients identified via the 'Endoscribe' database (n=195).<br><br>Characteristics not specified.<br><br>UK	To assess the sources of information for a group of patients with Barrett's oesophagus; the availability of Internet access for the patients and their views on the future availability and delivery of information.	People with oesophageal adenocarcinoma were excluded.
Griffiths 2011	Qualitative semi-structured interviews with framework for content analysis.	People at high risk of malignant progression from Barrett's columnar lined oesophagus enrolled in endoscopic surveillance program (N=22)<br><br>Aged 50-70 years	To explore patients' knowledge and understanding of Barrett's oesophagus and how the information forming the basis of that knowledge and understanding	Those with serious concomitant disease such as cancer, those who were unable to give valid consent, non-English speaking patients and those unable to read or write were excluded.

Supplementary Tables, di Pietro et al, National Institute for Health and Care Excellence (NICE) guidance on monitoring and management of Barrett's oesophagus and stage I oesophageal adenocarcinoma

Study	Design	Population	Research aim	Comments
		UK	influenced their self-management.	

Supplementary Tables, di Pietro et al, National Institute for Health and Care Excellence (NICE) guidance on monitoring and management of Barrett's oesophagus and stage I oesophageal adenocarcinoma

Supplementary Table 3: Summary of studies included in the evidence review for pharmacological interventions

Study	Intervention and comparison	Population	Outcomes	Comments
Babic 2015	PPI medication:<br><br>Pantoprazole (N = 54)<br>dose of 40mg twice a day during 10weeks<br><br>vs<br><br>Lansoprasole (N = 36)<br>dose of 30mg twice a day during 10 weeks, then 30mg once a day to the end of the study<br><br>vs<br><br>Omeprazole (N = 30)<br>dose of 40mg twice a day for 10weeks, then 40mg once a day	Patients with Barrett's oesophagus diagnosed by endoscopy and histological analysis of the tissue biopsy specimen<br><br>N=120<br>mean age (SD): 52.3 (14.4) years<br><br>Croatia	Indefinite dysplasia<br><br>Low-grade dysplasia<br><br>High-grade dysplasia<br><br>Follow up: 1 year	One patient in each Treatment group showed worsening and progression to higher grade of dysplasia at baseline.
Jankowski 2018	High or low dose PPI with or without aspirin.<br><br>High dose PPI: Esomeprazole (40 mg capsules twice daily; n=1270)<br>Vs<br>low dose (20 mg capsules once daily; n=1265).	People aged ≥18 years with circumferential Barrett's oesophagus of at least 1 cm in length (≥C1M1) or a tongue of Barrett's oesophagus of at least 2 cm in	All-cause mortality<br><br>Cause-specific mortality<br><br>High-grade dysplasia<br><br>Oesophageal adenocarcinoma	Participants in the AspECT trial were randomised using a 2x2 factorial design to receive high or low dose PPI with or

Supplementary Tables, di Pietro et al, National Institute for Health and Care Excellence (NICE) guidance on monitoring and management of Barrett's oesophagus and stage I oesophageal adenocarcinoma

Study	Intervention and comparison	Population	Outcomes	Comments
	Aspirin (300 mg in the UK, 325 mg in Canada; n=1138)<br>Vs<br>No aspirin (n=1142).<br><br>Study comparison groups:<br><br>1) High dose PPI vs low dose PPI (in each group there was an approximately equal number of people who did or did not receive aspirin)<br>2) Aspirin vs no aspirin (in each group there was an approximately equal number of people who received high and low dose PPI medication)	length (≥C0M2), irrespective of the presence now or historically of histologically proven intestinal metaplasia.<br><br>Countries: England, Scotland, Wales, and Northern Ireland, and one in McMaster Health Sciences Centre, Hamilton, ON, Canada	Serious adverse events(Blood and lymphatic system disorders, cardiac disorders, ear and labyrinth disorders, endocrine disorders, eye disorders, gastrointestinal disorders, general disorders and administration site conditions, hepatobiliary disorders, immune system disorders, infections and infestations, injury, poisoning, and procedural complications investigations, metabolism and nutrition disorders, musculoskeletal and connective tissue disorders, neoplasms benign, malignant, and unspecified (including cysts and polyps, nervous system disorders,	without aspirin.<br><br>Results were reported separately for the comparisons of low vs high dose PPI and aspirin vs no aspirin.

Supplementary Tables, di Pietro et al, National Institute for Health and Care Excellence (NICE) guidance on monitoring and management of Barrett's oesophagus and stage I oesophageal adenocarcinoma

Study	Intervention and comparison	Population	Outcomes	Comments
			psychiatric disorders, renal and urinary disorders, respiratory, thoracic, and mediastinal disorders, skin and subcutaneous tissue disorders, vascular disorders)<br><br>Follow up: Median 8.9 years	

Supplementary Tables, di Pietro et al, National Institute for Health and Care Excellence (NICE) guidance on monitoring and management of Barrett's oesophagus and stage I oesophageal adenocarcinoma

Supplementary Table 4: Summary of studies included in the evidence review on clinical effectiveness of endoscopic surveillance.

Study	Intervention and comparison	Population	Outcomes	Sub-group (adequate surveillance or non-adequate surveillance)	Comments
Corley, 2013	Surveillance endoscopy: any esophagogastroduodenoscopy performed principally for cancer surveillance of a previously documented Barrett's oesophagus, not for symptoms. (N=82)<br><br>Vs<br><br>No surveillance (N=57)	Cases: People diagnosed with oesophageal or gastroesophageal junction adenocarcinoma; with a Barrett's oesophagus diagnosis 6 months or more before their cancer diagnosis; who subsequently died of oesophageal/gastroesophageal junction adenocarcinoma or its complications(N=38).<br><br>Controls: had a diagnosis of Barrett's oesophagus who did not die of oesophageal or gastroesophageal junction adenocarcinoma through the end of the follow-up evaluation(N=101).<br><br>Controls were matched to cases by age at Barrett's oesophagus diagnosis, year of Barrett's oesophagus diagnosis, medical centre of Barrett's oesophagus diagnosis, sex, and race.	Mortality from OAC<br><br>At 3-year follow-up	Not possible to define as adequacy of surveillance not reported	Case control study<br><br>The cases and matched controls defined the outcome of oesophageal adenocarcinoma (OAC) mortality, and the association with prior surveillance was investigated with conditional logistic regression.<br><br>The logistic regression analysis only adjusted for dysplasia status but the controls were already fairly well-matched to the cases on the basis of a number of plausible potential confounders (see population column) so to some extent quite reasonable attempts were made to reduce selection bias.<br><br>The reasons for the comparator group not having surveillance was not explained.

Supplementary Tables, di Pietro et al, National Institute for Health and Care Excellence (NICE) guidance on monitoring and management of Barrett's oesophagus and stage I oesophageal adenocarcinoma

Study	Intervention and comparison	Population	Outcomes	Sub-group (adequate surveillance or non-adequate surveillance)	Comments
		Mean age at index date: surveillance group= 73.5 (8.2); no surveillance group=73.8 (8.1)<br><br>USA			
El-Serag, 2016	Barrett's Surveillance: included patients who received surveillance endoscopy for non-dysplastic Barrett's, or surveillance endoscopy for Barrett's with dysplasia initially detected as a result of Barrett's surveillance.<br><br>Vs<br><br>Comparator: Patients whose OAC was initially detected on diagnostic endoscopy, screening endoscopy, unknown indication for endoscopy or surveillance endoscopy for dysplasia originally detected in non-Barrett's surveillance endoscopy	Patients with Barrett's and OAC who were >18 years of age at Barrett's index date and had at least 1 year of follow-up after the index Barrett's endoscopy as well as before their last VA visit or date of oesophageal cancer(N=424).<br><br>Mean age (SD): 61.9 (9.9)<br><br>USA	Mortality from OAC<br>All-cause mortality<br><br>At 5-year follow-up	Not possible to define as adequacy of surveillance not reported	In this retrospective cohort study, a number of plausible covariates were included in the multivariable analyses, reducing to some extent the risk of selection bias. Adjustment was carried out for OAC diagnosis, age, ethnicity, propensity to go into the surveillance group, comorbidities, total number of VA/GI visits, stage and treatment.<br><br>The reasons for the comparator group not having surveillance was not explained.
MacDonald, 2000	Surveillance endoscopies: defined as examinations done only for surveillance. Endoscopies to investigate deteriorating symptoms	Surveillance group: People with proven Barrett's oesophagus who were potentially suitable for major surgery should a lesion be	Mortality from OAC<br>All-cause mortality	Not possible to define as adequacy of	This retrospective cohort study was at risk of critical bias. Firstly, the comparator group were systematically different to the

Supplementary Tables, di Pietro et al, National Institute for Health and Care Excellence (NICE) guidance on monitoring and management of Barrett's oesophagus and stage I oesophageal adenocarcinoma

Study	Intervention and comparison	Population	Outcomes	Sub-group (adequate surveillance or non-adequate surveillance)	Comments
	in a patient in the surveillance programme were not included as surveillance endoscopies.<br>Vs<br><br>No surveillance - this was in a group with Barrett's for whom surveillance was not regarded as appropriate. The main reasons were age >70 and co-existing serious illness.	detected, which usually meant patients younger than 70 who had no serious coexisting disease (N=143)<br><br>No surveillance group: were older (69 vs 57 years), less likely to be men (47% vs 60%), had a shorter length of metaplasia (73mm vs 81mm) and were less likely to have a stricture (5% vs 16%) compared to those in the surveillance group (N=266)<br><br>Mean age (range):<br>Surveillance group= 57 (17-69) years<br>No surveillance group= 69 (17-94) years<br><br>UK		surveillance not reported	surveillance group in prognostic characteristics. Secondly no statistical adjustments or matching were carried out.
Roberts, 2010	Scheduled annual endoscopy with 4 quadrant biopsies every 2cm<br><br>Vs	Patients with Barrett's metaplasia, and eventual OAC or HGD; fit for curative treatment (N=82)Mean age: not reported; age range: 46 to 93 years	All-cause mortality<br><br>At 5-year follow-up	Adequate: Four quadrant biopsies every 2cm	This retrospective cohort study was at risk of critical bias. Firstly, the comparator group were systematically different to the surveillance group in prognostic

Supplementary Tables, di Pietro et al, National Institute for Health and Care Excellence (NICE) guidance on monitoring and management of Barrett's oesophagus and stage I oesophageal adenocarcinoma

**Study**	**Intervention and comparison**	**Population**	**Outcomes**	**Sub-group (adequate surveillance or non-adequate surveillance)**	**Comments**
	Single endoscopy with no scheduled programme of surveillance	UK			characteristics. Secondly no statistical adjustments or matching were carried out.
Royston, 2016	Serial endoscopy and biopsy at 2-3-year intervals.<br><br>Vs<br><br>Clinical follow-up or lost to follow up	People with gastro-oesophageal reflux disease and Barrett's Oesophagus who later developed OAC (N=54)<br><br>UK	Mortality from OAC<br><br>At minimum 2-year follow-up	Not possible to define as adequacy of surveillance not reported	This retrospective cohort study was at risk of critical bias. Firstly, the comparator group were systematically different to the surveillance group in prognostic characteristics. Secondly no statistical adjustments or matching were carried out.
Theron, 2016	Endoscopic surveillance: people underwent endoscopic surveillance if suitable every 2 years until they reached 75 years of age or developed co-morbidity that, in the opinion of the responsible clinician, precluded further surveillance due to the risks of oesophagectomy.<br>Vs<br>No surveillance: Patients failing to attend surveillance endoscopy despite being clinically indicated for surveillance.	All patients diagnosed with Barrett's between 1982 and 2007 at City Hospital, Birmingham, and between 1997 and 2007 at the adjacent Sandwell General Hospital, West Bromwich (N=431)<br><br>Median age (range): endoscopic surveillance= 55.5 (51.2 to 66.6); no surveillance= 58 (49.2 to 63.6)<br><br>UK	Mortality from OAC<br>All-cause mortality<br>Progression to OAC<br><br>At 5-year follow-up	Adequate: Quadrantic biopsies were taken every 2 cm throughout the Barrett's segment in addition to targeted biopsies of any focal lesions and reported by a gastrointestinal histopathologist	This retrospective cohort study was also at risk of critical bias. Although the comparator group were clinically indicated for surveillance and did not receive it for non-clinical reasons, no attempt was made to adjust for any residual selection bias.
Verbeeck, 2014	Endoscopic surveillance: Participation in an endoscopic surveillance program was defined as a prior Barrett's diagnosis 1 year or	All patients diagnosed with OAC between 1999 and 2009 in the Netherlands were selected from	Mortality from OAC<br>Progression to type IV tumour stage	Not possible to define as adequacy of	This study looked at other groups which have not been included in this review as they are less relevant to the aims of the review:

Supplementary Tables, di Pietro et al, National Institute for Health and Care Excellence (NICE) guidance on monitoring and management of Barrett's oesophagus and stage I oesophageal adenocarcinoma

Study	Intervention and comparison	Population	Outcomes	Sub-group (adequate surveillance or non-adequate surveillance)	Comments
	longer before OAC diagnosis with at least one additional endoscopy with biopsies between the first histologic Barrett's and OAC diagnosis.<br><br>Vs<br><br>No surveillance: people with Barrett's not given any surveillance.	the nationwide Netherlands Cancer Registry (N=671)<br><br>Age details not specified.<br><br>Holland	At 2-year follow-up	surveillance not reported	inadequate surveillance and Barrett's unknown.<br><br>In this retrospective cohort study, a number of plausible covariates were included in the multivariable analysis, reducing to some extent the risk of selection bias in the mortality analysis. Adjustments were made for age, gender, time between Barretts diagnosis and OAC diagnosis, dysplasia grade, hospital type, tumour grade, tumour stage, resectability of tumour and treatment. However, the progression to type IV tumour stage analysis is unadjusted and therefore prone to high levels of selection bias, particularly since the reasons for the comparator group not having surveillance was not explained.

Supplementary Tables, di Pietro et al, National Institute for Health and Care Excellence (NICE) guidance on monitoring and management of Barrett's oesophagus and stage I oesophageal adenocarcinoma

Supplementary Table 5: Summary of studies included in the evidence review for diagnostic accuracy of endoscopic surveillance

Study	Population	Target condition	Index test	Reference standard	Comments
Chromoendoscopy					
Bajbouj 2010	Participants aged 18 – 80 years; Barrett's length at least COM1 according to Prague classification; in the case of suspected intraepithelial neoplastic changes, lesion <1cm; acid-suppressive therapy at least at the standard dose for a minimum of 4 weeks (n=68)<br><br>Age, mean (SD): 60 ± 12 years<br><br>Germany	Barrett's Oesophagus: high grade intraepithelial neoplasia / carcinoma	Probe based confocal laser endomicroscopy	Standard endoscopy	Diagnostic data reported per biopsy and per patient<br><br>2x2 data not reported
Canto 2014	Barrett's oesophagus patients undergoing routine surveillance or referred for confirmation of diagnosis and/or endoscopic therapy (n=192)<br><br>Median age (range): high-definition white-light endoscopy and random biopsy group: 62 (26 to 79); high-definition white-light endoscopy followed by laser endomicroscopy and targeted biopsy group: 62 (32 to 82)<br><br>USA	Barrett's oesophagus confocal neoplasia	High-definition white light endoscopy alone with random biopsies (HDWLE+RB)<br><br>High-definition white light endoscopy + endoscope-based confocal laser endomicroscopy (CLE) with targeted biopsies (HDWLE+CLE+TB)	Blinded expert pathologic diagnosis	Multi-centre RCT<br><br>2x2 data not reported

Supplementary Tables, di Pietro et al, National Institute for Health and Care Excellence (NICE) guidance on monitoring and management of Barrett's oesophagus and stage I oesophageal adenocarcinoma

Study	Population	Target condition	Index test	Reference standard	Comments
Curvers 2010	Patients with Barrett's oesophagus referred to 5 participating centres for work-up of endoscopically inconspicuous high-grade dysplasia/ early carcinoma (HGD/Ca) (n=87)<br><br>Age, mean (SD): 68 (9)<br><br>Netherlands & USA	Barrett's oesophagus with high grade dysplasia and early carcinoma	Endoscopic tri-modal imaging (incorporating high-resolution endoscopy, autofluorescence and narrow-band imaging)	Standard video endoscopy	Randomised cross-over multi-centre study<br><br>2x2 data calculated
Egger 2003	Participants undergoing routine surveillance for non dysplastic, dysplastic or first time in surveillance for confirmed Barrett's Oesophagus without (n=18) or with (n=8) only low grade dysplasia<br><br>Age, mean (range): 64.8 years; range 29–78<br><br>Germany	Barrett's Oesophagus with intestinal metaplasia with columnar and goblet cells vs low or high grade dysplasia, cancer	Autofluorescence<br><br>Methylene blue staining	Standard endoscopy	Diagnostic data given per biopsy and per patient<br><br>2x2 data not reported<br><br>Indirectness: sensitivity and specificity were not reported separately for dysplasia or cancer but also include metaplasia findings.
Jayasekera 2012	Patients referred for endoscopic evaluation and treatment of dysplastic Barrett's oesophagus, which had been previously diagnosed by their referring physician (n=50)<br><br>Age, median (range): 66 (41-86) years	Barrett's oesophagus with high grade dysplasia and intramucosal cancer.	Narrow-band imaging<br><br>Confocal laser endomicroscopy<br><br>High definition white light endoscopy	Histology (Seattle protocol)	Study aim: to assess 3 consecutive imaging modalities with histological assessment (standard Seattle protocol biopsies) as the reference standard.<br><br>2x2 data calculated

Supplementary Tables, di Pietro et al, National Institute for Health and Care Excellence (NICE) guidance on monitoring and management of Barrett's oesophagus and stage I oesophageal adenocarcinoma

Study	Population	Target condition	Index test	Reference standard	Comments
	Australia				Indirectness: Serious indirectness as results for white light endoscopy are given separately with biopsy as the reference standard
Longcroft-Wheaton 2020	People with biopsy-proven Barrett's oesophagus, no history or prior dysplasia or cancer, positive for intestinal metaplasia<br><br>Age, mean (SD): 66 (11.1)<br><br>UK	Barrett's oesophagus with neoplasia (high grade dysplasia, low grade dysplasia, cancer)	Acetic acid-targeted biopsies (Portsmouth protocol)	Seattle protocol-guided nontargeted biopsies.	Pilot multi-centre randomised cross-over trial<br><br>2x2 data calculated
Ormeci 2008	Patients older than 18 years with an indication for esophagogastroduodenoscopy were selected for this study (n=109)<br><br>Age, mean (SD): 62.32 (10.61 years); range, 33–82 years<br><br>Turkey	Barrett's Oesophagus with dysplasia or cancer	Chromoendoscopy with methylene blue	Standard endoscopy	Histopathologic diagnosis was accepted as the gold standard, and conventional endoscopic or chromoendoscopic diagnosis was compared with the histopathologic diagnosis.<br><br>Results from chromoendoscopy and standard/conventional endoscopy reported separately.<br><br>2x2 data not reported
Pascarenco 2016	Patients over 18 with endoscopic confirmation of Barrett's Oesophagus (n=84)<br><br>Age, mean (range): 57.4 (26-84)	Barrett's oesophagus with low grade dysplasia or indefinite for dysplasia	Narrow-band imaging	White light imaging	2x2 data calculated

Supplementary Tables, di Pietro et al, National Institute for Health and Care Excellence (NICE) guidance on monitoring and management of Barrett's oesophagus and stage I oesophageal adenocarcinoma

Study	Population	Target condition	Index test	Reference standard	Comments
	Romania				
Ragunath 2003	Patients with endoscopic and histological diagnosis of Barrett's oesophagus segments of 3cm or more in length, adults patients of any sex attending for endoscopy, including newly diagnosed patients as well as those undergoing surveillance endoscopy for Barrett's Oesophagus, and patients known to have dysplasia without mucosal abnormalities who were receiving follow up endoscopies (n=57)<br><br>Age: not reported<br><br>UK	Barrett's Oesophagus with dysplasia or carcinoma	Methylene blue	Standard endoscopy	2x2 data not reported
Sharma 2011	Consecutive patients undergoing BE surveillance and/or referred for BE-associated neoplasia (HGD/oesophageal carcinoma) evaluation and treatment were prospectively enrolled in this trial at 5 hospitals (n=101)<br><br>Age, mean (range): 65.1 years (27–90 years)<br><br>France, Germany & USA	Barrett's Oesophagus: high grade dysplasia / oesophageal cancer	Narrow-band imaging<br><br>Probe-based confocal laser endomicroscopy	Histology	Diagnostic data reported per location<br><br>2x2 data calculated<br><br>Indirectness: the paper measures diagnostic accuracy of the visual findings from each HD-WLE, NBI, pCLE with reference to the full histological findings. i.e. reference standard was histology derived from biopsies from each

Supplementary Tables, di Pietro et al, National Institute for Health and Care Excellence (NICE) guidance on monitoring and management of Barrett's oesophagus and stage I oesophageal adenocarcinoma

Study	Population	Target condition	Index test	Reference standard	Comments
					procedure rather than histology from biopsies from the HD-WLE
Sharma 2013	Patients undergoing screening or surveillance for Barrett's oesophagus at three tertiary referral centres.<br><br>Age, mean (range): 61 (38-85) years<br><br>USA, Netherlands	Barrett's oesophagus with neoplasia (high grade dysplasia, oesophageal adenocarcinoma)	Narrow-band imaging	White-light endoscopy	Multi-centre randomised cross-over trial<br><br>2x2 data calculated
Vithayathil 2022	Non-dysplastic Barrett's oesophagus patients (n=134)<br><br>Age, median (range): 67.3 (38.0 to 89.0) years<br><br>UK	Dysplasia (dysplasia and high-grade dysplasia)	Autofluorescence imaging- guided probe-based confocal laser endomicroscopy and molecular biomarkers (3-biomarker panel) (AFI-guided pCLE)<br><br>High-resolution white-light endoscopy with Seattle protocol biopsies	Histology	Cross-over RCT<br><br>Biomarkers: p53 and cyclin A by immunohistochemistry; aneuploidy by image cytometry)
<u>Endoscopic Brushing</u>					
Anandasabapathy 2011	Subjects with a known prior history (recent or remote) of	Barrett's Oesophagus: Barrett's metaplasia	Brush biopsy	Forceps biopsy (refers to Seattle protocol biopsy)	Study does not mention the type or methodology of endoscopic

Supplementary Tables, di Pietro et al, National Institute for Health and Care Excellence (NICE) guidance on monitoring and management of Barrett's oesophagus and stage I oesophageal adenocarcinoma

Study	Population	Target condition	Index test	Reference standard	Comments
	Barrett's oesophagus with dysplasia/neoplasia (indefinite for-dysplasia (IND), low-grade (LGD), high-grade dysplasia (HGD) or intramucosal adenocarcinoma (IMCA) and no grossly evident lesion (n=181)<br><br>Age, mean (range): 65 (46 – 87)<br><br>USA	(IM), indefinite for dysplasia (IND), dysplasia (LGD/ HGD/CA), and inadequate (no Barrett's oesophagus)			examination for biopsies and only notes the comparison of brush versus forceps.<br><br>2x2 data available
Artificial Intelligence					
Ebigbo 2021	Endoscopic, high resolution, white light images of T1a and T1b Barett's Cancer were collected retrospectively in three tertiary care centres in Germany (n=230 images)<br><br>Age not reported<br><br>Germany	Barrett's Oesophagus with T1a or T1b neoplasia	Convolutional neural networks	Histopathology (from white light imaging samples)	2x2 data not reported
Hashimoto 2020	Images from participants with histologically proven dysplasia (high grade dysplasia and T1 adenocarcinoma) in Barrett's (n=100 patients; 1832 images)<br><br>Age: not reported	Barrett's Oesophagus with high grade dysplasia	Narrow-band imaging + AI	White light imaging	Results for: narrow-band imaging +AI and white light imaging + AI, are provided separately with histology used as the reference standard<br><br>Diagnostic data given per image taken

Supplementary Tables, di Pietro et al, National Institute for Health and Care Excellence (NICE) guidance on monitoring and management of Barrett's oesophagus and stage I oesophageal adenocarcinoma

Study	Population	Target condition	Index test	Reference standard	Comments
	USA				2x2 data calculated

Supplementary Tables, di Pietro et al, National Institute for Health and Care Excellence (NICE) guidance on monitoring and management of Barrett's oesophagus and stage I oesophageal adenocarcinoma

Supplementary Table 6: Summary of studies included in the evidence review for staging of oesophageal adenocarcinoma

Study	Population	Target condition	Index test	Reference standard	Comments
Cen 2008	Oesophageal cancer patients (N=87; n=81 with adenocar-cinoma, n=6 squamous cell carcinoma)<br><br>Median age: 65 years<br><br>USA	T1 staging (T1a vs T1b, T2-4)<br><br>Metastasis/N staging (N0 vs N1)	EUS	Histopathologic assessment of resected specimens	Retrospective study<br><br>Indirectness: N=22 had cancer above stage 1 but it is not clear if this was suspected from baseline.<br><br>N=6 had squamous cell carcinoma
Pech 2006	Patients with confirmed 'early' cancer in Barrett's oesophagus (n=100)<br><br>Median age (range): 64 (58-72) years<br><br>The T category was assessed using high frequency probes (HFPs) in 66/100	T (T1m vs T1sm) and N staging<br><br>Staging using the Paris classification<br><br>T1m assumed to correspond to T1a<br>T1sm assumed to correspond to T1b	(Miniprobe) EUS (upper gastrointestinal endoscopy)<br><br>CT (of the chest and upper abdomen, and abdominal ultrasonograph y)	Histology (based on endoscopic resection or surgical specimens)	Patients in whom carcinoma could not be confirmed by experienced gastroenterologica l pathologists were excluded<br><br>Includes people with T2 (n=4) and T3(n=3) histology; data for EUS calculated including those with T1m and T1sm staging based on EUS and

Supplementary Tables, di Pietro et al, National Institute for Health and Care Excellence (NICE) guidance on monitoring and management of Barrett's oesophagus and stage I oesophageal adenocarcinoma

Study	Population	Target condition	Index test	Reference standard	Comments
	patients who had elevated and/or depressed lesions. EUS with HFPs was not carried out on endoscopically unequivocal mucosal neoplasia (type IIb lesions in the Paris classification)<br><br>Germany				Histology (n=55), excluding T2 and T3<br><br>2x2 table for N staging and T staging for CT could not be calculated from the paper.
Pech 2010	Patients with suspected 'early' cancer in Barrett's oesophagus, referred for endoscopic treatment for Barrett's cancer (n=43)<br><br>Median age (range): 66 (58-73)<br><br>Germany	T staging (mucosal and submucosal Barrett's cancer i.e. T1m vs T1sm)<br><br>N staging (N1, N0)	High frequency mini-probes (HFPs)<br><br>Conventional radial endoscopic ultrasonograph y (crEUS)	Histology (based on endoscopic resection or surgical specimens)	Prospective randomised cross-over study<br><br>Includes people with macroscopic tumour type: I-III; with histological stage T1m1-4 and T1sm1-3<br><br>2x2 table only calculated for N staging

Supplementary Tables, di Pietro et al, National Institute for Health and Care Excellence (NICE) guidance on monitoring and management of Barrett's oesophagus and stage I oesophageal adenocarcinoma

Study	Population	Target condition	Index test	Reference standard	Comments
Scotiniotis 2001	Patients with Barrett's oesophagus and high-grade dysplasia or intramucosal carcinoma based on endoscopy, endoscopic biopsies, and CT. N=22<br><br>Mean age (SD) 64 (8.7) years<br><br>USA	T staging (T1b vs Tcis/T1a, T2, T3)	EUS	Surgical/pathologic evaluation	Retrospective study<br><br>Indirectness: n=4 had cancer at stages T2 and T3<br><br>N staging results not included in the present review as 3/5 people classified as positive of lymph node metastasis by the EUS had stage beyond T1 (T2/3) and therefore the majority of people for which sensitivity results were based did not meet the review protocol.
Thomas 2010	Patients with histologically proven high-grade intraepithelial neoplasia or	T staging (; T1sm vs T0 and T1m)<br><br>N staging (N1 vs N0)	EUS	Histology	Retrospective study<br><br>Pre-EUS histology based on 1 to 2 esophagogastruodenoscopies and 2

Supplementary Tables, di Pietro et al, National Institute for Health and Care Excellence (NICE) guidance on monitoring and management of Barrett's oesophagus and stage I oesophageal adenocarcinoma

Study	Population	Target condition	Index test	Reference standard	Comments
	intramucosal carcinoma (n=50)<br><br>Median age (range): 69 (60-79) years<br><br>UK				to 36 mucosal biopsies (median 12): n=31 (62%) high-grade dysplasia, n=10 (38%) intramucosal carcinoma

Supplementary Tables, di Pietro et al, National Institute for Health and Care Excellence (NICE) guidance on monitoring and management of Barrett's oesophagus and stage I oesophageal adenocarcinoma

Supplementary Table 7: Diagnostic accuracy evidence for staging for suspected stage 1 oesophageal adenocarcinoma

Studies	N	Risk of bias	Inconsistency	Indirectness	Imprecision	Effect size (95%CI)	Quality
CT to detect T1 tumours in people with 'early' cancer in Barrett's oesophagus							
1 prospective cohort study	66	Serious[1]	Not serious	Serious[2]	Cannot be assessed[3]	Sensitivity=1.00	VERY LOW
		Serious[1]	Not serious	Serious[2]	Cannot be assessed[3]	Specificity=0.00	VERY LOW
Mini-probe EUS to detect T1a vs T1b ('T1m vs T1sm') in people with 'early' cancer in Barrett's oesophagus							
1 prospective cohort study	55	Serious[1]	Not serious	Serious[2]	Serious[4]	Sensitivity= 0.89 (0.75 -0.96)	VERY LOW
		Serious[1]	Not serious	Serious[2]	Serious[4]	Specificity= 0.27 (0.06 -0.61)	VERY LOW
CT to detect N staging in people with 'early' cancer in Barrett's oesophagus							
1 prospective cohort study	66	Serious[1]	Not serious	Serious[2]	Cannot be assessed[3]	Sensitivity= 0.38	VERY LOW
		Serious[1]	Not serious	Serious[2]	Cannot be assessed[3]	Specificity=1.00	VERY LOW
Mini-probe EUS to detect N staging in people with 'early' cancer in Barrett's oesophagus							
1 prospective cohort study	66	Serious[1]	Not serious	Serious[2]	Cannot be assessed[3]	Sensitivity= 0.75	VERY LOW
		Serious[1]	Not serious	Serious[2]	Cannot be assessed[3]	Specificity= 0.97	VERY LOW
HFPs for T1a ('pT1m') in people with suspected 'early' cancer in Barrett's oesophagus							
1 prospective randomised cross-over study	36	Serious[1]	Not serious	Serious[2]	Serious[4]	Sensitivity= 0.70 (0.46 -0.87)	VERY LOW
	36	Serious[1]	Not serious	Serious[2]	Very serious[4]	Specificity= 0.69 (0.41- 0.88)	VERY LOW
HFPs for T1b ('pT1sm') in people with suspected 'early' cancer in Barrett's oesophagus							
1 prospective randomised cross-over study	36	Serious[1]	Not serious	Serious[2]	Serious[4]	Sensitivity= 0.69 (0.41 -0.88)	VERY LOW
		Serious[1]	Not serious	Serious[2]	Serious[4]	Specificity= 0.75 (0.50 -0.90)	VERY LOW
crEUS for T1a ('pT1m') in people with suspected 'early' cancer in Barrett's oesophagus							

Supplementary Tables, di Pietro et al, National Institute for Health and Care Excellence (NICE) guidance on monitoring and management of Barrett's oesophagus and stage I oesophageal adenocarcinoma

Studies	N	Risk of bias	Inconsistency	Indirectness	Imprecision	Effect size (95%CI)	Quality
1 prospective randomised cross-over study	25	Serious[1]	Not serious	Serious[2]	Very serious[4]	Sensitivity= 0.73 (0.39 -0.93)	VERY LOW
		Serious[1]	Not serious	Serious[2]	Very serious[4]	Specificity= 0.78 (0.49- 0.94)	VERY LOW
crEUS for T1b ('pT1sm') in people with suspected 'early' cancer in Barrett's oesophagus							
1 prospective randomised cross-over study	25	Serious[1]	Not serious	Serious[2]	Serious[4]	Sensitivity= 0.64 (0.36- 0.86)	VERY LOW
		Serious[1]	Not serious	Serious[2]	Very serious[4]	Specificity= 0.73 (0.39- 0.93)	VERY LOW
crEUS for N1 status in people with suspected 'early' cancer in Barrett's oesophagus							
1 prospective randomised cross-over study	16	Serious[1]	Not serious	Serious[2]	Very serious[4]	Sensitivity= 1.00 (0.29- 1.00)	VERY LOW
		Serious[1]	Not serious	Serious[2]	Serious[4]	Specificity= 0.92 (0.64- 1.00)	VERY LOW
EUS for T1a (vs T1b, T2-4) in people with oesophageal cancer							
1 retrospective cohort study	87	Serious[1]	Not serious	Very serious[2]	Serious[4]	Sensitivity= 0.67 (0.50- 0.80)	VERY LOW
		Serious[1]	Not serious	Very serious[2]	Serious[4]	Specificity= 0.93 (0.82- 0.99)	VERY LOW
EUS for N1 (vs N0) in people with oesophageal cancer							
1 retrospective cohort study	87	Serious[1]	Not serious	Very serious[2]	Serious[4]	Sensitivity= 0.38 (0.18- 0.62)	VERY LOW
		Serious[1]	Not serious	Very serious[2]	Not serious	Specificity= 0.94 (0.85- 0.98)	VERY LOW
EUS for T1b (vs T0, T1a) in people with high-grade intraepithelial neoplasia or intramucosal carcinoma							
1 retrospective series	46	Very serious[1]	Not serious	Not serious	Serious[4]	Sensitivity= 0.56 (0.31- 0.78)	VERY LOW
		Very serious[1]	Not serious	Not serious	Serious[4]	Specificity: 0.93 (0.76- 0.99)	VERY LOW
EUS for N1 status in people with high-grade intraepithelial neoplasia or intramucosal carcinoma							
1 retrospective series	29	Very serious[1]	Not serious	Not serious	Very serious[4]	Sensitivity= 0.50 (0.01- 0.99)	VERY LOW
		Very serious[1]	Not serious	Not serious	Serious[4]	Specificity= 0.96 (0.81- 1.00)	VERY LOW
EUS for T1b (Tcis/T1a, T2, T3) in people with high-grade dysplasia or intramucosal carcinoma							
1 retrospective cohort study	22	Serious[1]	Not serious	Serious[2]	Serious[4]	Sensitivity: 1.00 (0.48- 1.00)	VERY LOW
		Serious[1]	Not serious	Serious[2]	Serious[4]	Specificity: 0.94 (0.71- 1.00)	VERY LOW

Supplementary Tables, di Pietro et al, National Institute for Health and Care Excellence (NICE) guidance on monitoring and management of Barrett's oesophagus and stage I oesophageal adenocarcinoma

[1] Risk of bias was assessed using the QUADAS-2 checklist. The evidence was downgraded by 1 increment if the studies were rated at serious risk of bias and downgraded by 2 increments if the studies were rated at very serious risk of bias.

[2] Evidence was downgraded by 1 increment due to serious concerns over population indirectness or by 2 increments due to very serious concerns over population indirectness.

[3] Where the study does not report confidence intervals or the data to calculate 2x2 tables imprecision cannot be assessed. Where this is the case evidence quality was downgraded by 1 increment.

[4] Imprecision was assessed based on inspection of the confidence intervals. For sensitivity, two clinical decision thresholds were determined at the value above which a test would be recommended (90%), and a second below which a test would be considered of no clinical use (60%). For specificity, two clinical decision thresholds were determined at the value above which a test would be recommended (80%), and a second below which a test would be considered of no clinical use (50%). The evidence was downgraded by 1 increment when the range of the confidence interval around the point estimate crossed one threshold and downgraded by 2 increments when the range covered two thresholds.

Supplementary Tables, di Pietro et al, National Institute for Health and Care Excellence (NICE) guidance on monitoring and management of Barrett's oesophagus and stage I oesophageal adenocarcinoma

Supplementary Table 8: Summary of studies included in the evidence review for treatment of low-grade dysplasia

Study	Intervention and comparison	Population	Outcomes	Comments
Barret 2021	Radiofrequency ablation (RFA) (n=40)<br><br>vs<br><br>Endoscopic surveillance: annually (n=42)	Barrett's oesophagus patients with confirmed low-grade dysplasia (n=82)<br><br>Mean age (SD): 62.3 (10.06) years<br><br>France	Complete eradication of dysplasia<br><br>Complete eradication of intestinal metaplasia<br><br>Persistent low-grade dysplasia at 3 years (extracted as proxy for recurrence of dysplasia)<br><br>Complications (including fever, chest pain, upper GI bleeding, stricture)<br><br>Progression to high-grade dysplasia or adenocarcinoma<br><br>3 years after randomisation	Multicentre RCT (14 French centres)<br><br>N=81 (98.8%) were on PPIs at inclusion<br><br>Anti-reflux surgery had been performed in n=16 patients (19.5%).<br><br>N=8 (9.8%) had prior endoscopic resection for high-grade dysplasia/ early adenocarcinoma.

Supplementary Tables, di Pietro et al, National Institute for Health and Care Excellence (NICE) guidance on monitoring and management of Barrett's oesophagus and stage I oesophageal adenocarcinoma

Study	Intervention and comparison	Population	Outcomes	Comments
Phoa 2014	RFA (n=68); (double-dose PPI was given as maintenance therapy during the trial)<br><br>vs<br><br>Endoscopic surveillance (n=68):	Barrett's oesophagus patients with low-grade dysplasia (n=136)<br><br>Mean age (SD): 63 (9.51) years<br><br>The Netherlands, Belgium, UK, Ireland, Germany	Complete eradication of dysplasia<br><br>Complete eradication of intestinal metaplasia<br><br>Progression to high-grade dysplasia/ adenocarcinoma<br><br>Adverse events (protocol outcome: complications)<br><br>During a 3-year follow-up	Multicentre RCT (9 European centres): the Surveillance vs Radiofrequency Ablation (SURF) study)<br><br>The trial was terminated early due to the superiority of ablation for the primary outcome (neoplastic progression) and the potential for safety issues if the trial continued.<br>At point of termination, participants had completed at least 2 years of follow-up.
Pouw 2020	RFA (n=68)<br><br>Vs<br><br>Endoscopic surveillance (n=68)	Barrett's oesophagus patients with low-grade dysplasia (n=136)	Progression to high-grade dysplasia/ adenocarcinoma<br><br>Follow-up: 73 months; additional median follow-up	Retrospective cohort study of patients included in the SURF study.<br><br>Further non-comparative data available: at long-term follow-up 15 patients

Supplementary Tables, di Pietro et al, National Institute for Health and Care Excellence (NICE) guidance on monitoring and management of Barrett's oesophagus and stage I oesophageal adenocarcinoma

Study	Intervention and comparison	Population	Outcomes	Comments
		The Netherlands, Belgium, UK, Ireland, Germany	of 40 months (IQR 12-51) of the SURF study.	from the surveillance group were offered RFA. Complete clearance of intestinal metaplasia was found in 75/83 patients and recurrence was found in 7/75.
Shaheen 2009	RFA<br><br>Vs<br><br>Sham endoscopic procedure	Patients with dysplastic Barrett's oesophagus (n=127; n=64 had low-grade dysplasia and were included in this review)<br><br>Mean age (range): 65.72 (41-78)<br><br>USA	Complete eradication of dysplasia<br><br>Complete eradication of intestinal metaplasia<br><br>Progression to high-grade dysplasia/cancer<br><br>At 12 months	Multicentre RCT (19 sites)<br><br>Includes people with high-grade dysplasia but randomisation and results were stratified by grade of dysplasia; only results relevant to the low-grade dysplasia population are presented in the present review.

Supplementary Tables, di Pietro et al, National Institute for Health and Care Excellence (NICE) guidance on monitoring and management of Barrett's oesophagus and stage I oesophageal adenocarcinoma

Supplementary Table 9: Summary of studies included in the evidence review for treatment of high-grade dysplasia and stage I adenocarcinoma

Study	Intervention and comparison	Population	Outcomes	Comments
Li 2016	EMR+RFA (n=406)<br><br>Vs<br><br>RFA alone (n=857)	Patients with high-grade dysplasia (n=1054) or intramucosal carcinoma (IM) (n=209); Total n=1263<br><br>Mean age (SD): 66.59 (10.34) years<br><br>USA	Treatment related mortality<br><br>Complete eradication of dysplasia<br><br>Complete eradication of intestinal metaplasia<br><br>Recurrence of Intestinal metaplasia<br><br>Complications of treatment (stricture, GI bleeding, hospitalisation)<br><br>Number of RFA sessions required (protocol outcome: need for treatment)	US RFA Patient Registry<br><br>Retrospective observational study; multicentre registry including people who had RFA preceded by EMR.<br><br>Patients with EMR before RFA had worse pre-treatment histology (IMC, 38% vs 6%), shorter BE segment (mean 4.6 vs 5.4 cm) and were less likely to be taking twice-daily PPIs (74% vs 81%)<br><br>Outcomes stratified by baseline grade of dysplasia

Supplementary Tables, di Pietro et al, National Institute for Health and Care Excellence (NICE) guidance on monitoring and management of Barrett's oesophagus and stage I oesophageal adenocarcinoma

Study	Intervention and comparison	Population	Outcomes	Comments
			At mean (SD) follow-up time: 2.86 (1.53) years for EMR+RFA, 2.76 (1.66) years for RFA alone	
Manner 2014	APC (n=33)<br><br>Vs<br><br>Surveillance (n=30)<br><br>PPI (esomeprazole) was administered in both treatment groups (dosage adjusted to the patients' 24-hour PH-metry finding 40 or 80 mg per day)	Patients in whom focal early Barrett's neoplasia (high-grade neoplasia, n=40 or mucosal cancer, n=23) had been curatively resected by endoscopy (n=63)<br><br>Mean age (SD; range): 63 (1; 42-79) years<br><br>Germany	Recurrence (of neoplasia)<br><br>2-year follow-up; Mean follow-up (SD, range): ablation group= 28.2 (13.7, 0-44) months; surveillance group= 24.7 (14.8; 0-45) months	RCT
Peerally 2019	ER + APC (n=40)<br><br>Vs<br><br>ER+ RFA (n=36)<br><br>High-dose PPI (twice daily) was	Patients with high-grade dysplasia (n=58) or T1a cancer (n=18); Total n=76<br><br>Mean age: 69.7 years	Clearance of high-grade dysplasia/cancer<br><br>Clearance of Barrett's oesophagus	Multicentre pilot RCT: Barrett's Randomised Intervention for Dysplasia by Endoscopy (BRIDE study); 6 tertiary-care referral centres

Supplementary Tables, di Pietro et al, National Institute for Health and Care Excellence (NICE) guidance on monitoring and management of Barrett's oesophagus and stage I oesophageal adenocarcinoma

Study	Intervention and comparison	Population	Outcomes	Comments
	administered to all patients.	UK	Adverse events (stricture, GI bleeding) (protocol outcome: complications of treatment)<br><br>12 months	N=65 completed the trial<br><br>Study also reports QoL (EQ-5D, QLQ-C30, chest-pain, dysphagia) but not in an extractable format: graph format with no specific scores reported
Pouw 2011	ER-cap; n=42<br><br>Vs<br><br>ER with Multi-band mucosectomy (MBM); n=42	Patients with Barrett's oesophagus with biopsy proven high-grade dysplasia (n=19) and/or early cancer (n=52); Total n=84<br><br>Median age (IQR): 70 (63.3-76) years<br><br>The Netherlands	Complications of treatment: bleeding, perforation<br><br>During the procedure and 0-48 hours later.	RCT<br><br>Perforations occurring in the ER-cap group were reported as moderate; perforation occurring in the MBM group was reported as severe<br><br>Complication severity: moderate (4-10 days hospitalisation, need for repeat endoscopic intervention), severe (>10 days of hospitalisation, intensive care unit admission, need for

Supplementary Tables, di Pietro et al, National Institute for Health and Care Excellence (NICE) guidance on monitoring and management of Barrett's oesophagus and stage I oesophageal adenocarcinoma

Study	Intervention and comparison	Population	Outcomes	Comments
				surgery), fatal (death attributable to procedure <30 days or longer with continuous hospitalization)
Shaheen 2009	RFA<br><br>Vs<br><br>Sham endoscopic procedure<br><br>All patients received 40 mg of esomeprazole twice daily throughout the trial	Patients with dysplastic Barrett's oesophagus (n=127; n=63 had high-grade dysplasia and were included in this review)<br><br>Mean age (range): 66.37 (49-80)<br><br>USA	Complete eradication of dysplasia<br><br>Complete eradication of intestinal metaplasia<br><br>At 12 months	Multicentre RCT (19 sites)<br><br>Includes people with low-grade dysplasia but randomisation and results were stratified by grade of dysplasia; only results relevant to the high-grade dysplasia population are presented in the present review.
Terheggen 2017	ESD (n=20)<br><br>Vs<br><br>EMR (n=20)<br><br>PPI was orally administered in double standard during the study period.	Barrett's oesophagus patients with high-grade intraepithelial neoplasia (HGIN, N=9) or early adenocarcinoma (EAC) (n=31); Total n=40	Complete resection of high-grade intraepithelial neoplasia or adenocarcinoma<br><br>Curative resection (histologically complete resection of HGIN/ mucosal EAC or EAC with	RCT<br><br>Pre-treatment histology:<br><br>ESD: n=5 high-grade intraepithelial neoplasia, n=15 adenocarcinoma; EMR: n=4 high-grade intraepithelial

Supplementary Tables, di Pietro et al, National Institute for Health and Care Excellence (NICE) guidance on monitoring and management of Barrett's oesophagus and stage I oesophageal adenocarcinoma

Study	Intervention and comparison	Population	Outcomes	Comments
		Mean age (SD): 64.5 (11.52) years<br><br>Germany	low-risk superficial submucosal invasion)<br><br>Adverse events (perforation, mediastinitis, temporary chest discomfort, severe adverse events)<br><br>Up to 30 days after the procedure<br><br>Complete remission of neoplasia after initial resection<br><br>Complete remission of intestinal neoplasia<br><br>Recurrent neoplasia<br><br>Conversion of endoscopic treatment to surgery (Referral to elective surgery)	neoplasia, n=16 adenocarcinoma<br><br>Severe adverse events: that caused prolongation of hospitalisation and/or its management required additional therapeutic interventions, 30-day mortality

Supplementary Tables, di Pietro et al, National Institute for Health and Care Excellence (NICE) guidance on monitoring and management of Barrett's oesophagus and stage I oesophageal adenocarcinoma

Study	Intervention and comparison	Population	Outcomes	Comments
			>30 day follow-up (mean (SD) follow-up was 22.6 (7.8) months for the ESD and 23.6 (5) months for the EMR group.	
Thota 2018	RFA (n=73)<br><br>Vs<br><br>Cryotherapy (cryo-spray; n=81)	Barrett's oesophagus patients with dysplasia or intramucosal carcinoma; N=154<br><br>USA	Mortality (all-cause and disease specific)<br><br>Complete eradication of intestinal metaplasia<br><br>Complete eradication of dysplasia<br><br>Recurrence<br><br>2-year follow up	Retrospective observational study<br><br>Indirectness: Includes 23/154 (15%) had low-grade dysplasia at baseline
van Vilsteren 2011	Focal ER (ER-cap technique) + Stepwise radical ER (SRER) (n=25)	Barrett's oesophagus patients with high-grade dysplasia	Complete histological response for	Multi-centre RCT (3 centres)

Supplementary Tables, di Pietro et al, National Institute for Health and Care Excellence (NICE) guidance on monitoring and management of Barrett's oesophagus and stage I oesophageal adenocarcinoma

Study	Intervention and comparison	Population	Outcomes	Comments
	Vs<br><br>Focal ER (ER-cap technique) + RFA (n=22)	(n=19) or early cancer (n=28); Total n=55<br><br>Median age (range): 68 (45-88) years<br><br>Germany, The Netherlands	neoplasia (CR-neoplasia)<br><br>Complete histological response for intestinal metaplasia (CR-IM)<br><br>Recurrence<br><br>Complications (severe, moderate, mild)<br><br>Median (IQR) follow-up from initial treatment 24 (18-29) months; from final treatment sessions 18 (11-23) months	Recurrence notes was of early cancer, requiring ER.

Supplementary Tables, di Pietro et al, National Institute for Health and Care Excellence (NICE) guidance on monitoring and management of Barrett's oesophagus and stage I oesophageal adenocarcinoma

Supplementary Table 10: Summary of studies included in the evidence review comparing oesophagectomy and endoscopic resection for treatment of stage I adenocarcinoma

Study (n)	Intervention	Comparator	Inclusion/exclusion criteria	Stage	Grade of differentiation	LVI	R0/R1	Submucosal grades
Li, 2017<br>N=23	oesophagectomy was performed by Ivor-Lewis or transhiatal technique	Endoscopic mucosal resection (EMR)	Inclusion: Patients with intramucosal carcinoma and Barrett's oesophagus from a single-centre pathology specimen database; undergoing EMR or oesophagectomy; Adenocarcinoma<br>Exclusion: Patients with invasive disease not amenable to endoscopic treatment (deeper than T1sm1 or node positive) were excluded	All T1a or T1b. T1a 92% in Sx group and 91% in endo group.<br>Taken as T1a overall	Mostly well differentiated (67% Sx, 82% endo)	none	No data	Only 2 submucosal cases: both sm1.
Pacifico, 2003<br>N=88	Oesophagectomy – no details were provided.	EMR followed by photodynamic therapy (PDT) about 1 month later	Inclusion: All patients with Barretts Oesophagus and adenocarcinoma. The stage of adenocarcinoma was determined by EUS and histology of the EMR specimen if surgery was not performed and by histopathologic staging following esophagectomy. Patients who were considered to have mucosal disease by EUS and/or found to be surgical stage 0	62/64 T1 in Sx group, and 24/24 T1 in endo group.<br>Taken as 'mixed' T1 as no sub-grouping for T1a and T1b.	No data	No data	No data	No data

Supplementary Tables, di Pietro et al, National Institute for Health and Care Excellence (NICE) guidance on monitoring and management of Barrett's oesophagus and stage I oesophageal adenocarcinoma

Study (n)	Intervention	Comparator	Inclusion/exclusion criteria	Stage	Grade of differentiation	LVI	R0/R1	Submucosal grades
			or 1 were included in the study.<br>Exclusion: Patients with lymph node metastasis					
Pech, 2011<br>N=114	Transthoracic oesophageal resection. En-bloc oesophagectomy after open or laparoscopic gastric mobilisation	EMR. After endoscopic resection, following later follow up and remission of neoplasia, patients underwent ablation of the remaining nondysplastic epithelium using argon plasma coagulation	Inclusion: Patients with mucosal Barretts adenocarcinoma treated with surgery or endoscopy.<br>Exclusion: Patients with ypT1 after neoadjuvant chemoradiation	All T1a	Most graded 1 or 2: 36/38 Sx group, 72/74 endo group	No lymph or vascular invasion	No data	Not applicable
Prasad, 2009<br>N=178	Oesophagectomy was via the transthoracic or the transhiatal route.	The initial technique was a variceal ligation method in which a Bard Six-Shooter and suction was used to retract the lesion of interest and had	Inclusion: Patients were either referred for endoscopic treatment of mucosal EAC to the Barrett's oesophagus Unit by physicians or were under surveillance for HGD in the BE Unit. All patients seen in the BE Unit for endoscopic therapy had either received	All T1a	No data	4 in Sx group had metastatic lymphadenopathy; none in endo gp	No data	Not applicable

Supplementary Tables, di Pietro et al, National Institute for Health and Care Excellence (NICE) guidance on monitoring and management of Barrett's oesophagus and stage I oesophageal adenocarcinoma

Study (n)	Intervention	Comparator	Inclusion/exclusion criteria	Stage	Grade of differentiation	LVI	R0/R1	Submucosal grades
		a band placed over it to create a pseudopolyp, which was then resected. Beginning in April 2000, EMR was performed. PDT was administered at a lter date after the achievement of histologic remission.	consultation with thoracic surgeons at the Mayo Clinic or at their local hospitals. Patients referred for esophagectomy were usually referred directly by their physicians or were elected to undergo surgery after initial evaluation at the BE Unit. Barrett's oesophagus and mucosal OAC<br>Exclusion: Submucosal carcinoma					
Schmidt, 2016<br>N=85	Oesophagectomy - surgical patients underwent transthoracic oesophageal resections in 94 % and transhiatal resections in 6 %.	Endoscopic treatments - in patients undergoing endoscopic therapy, all visible neoplastic lesions were treated with EMR. Residual Barrett's oesophagus mucosa was subsequently treated with	Inclusion: All Barrett's patients presenting with clinically T1a OAC; undergone oesophageal resection or endoscopic treatment of Barrett's oesophagus<br>Exclusion: Patients with high-grade dysplasia, submucosal invasion (T1b EAC), and those undergoing neoadjuvant therapy.	T1a	No data	4 with nodal mets in Sx group; 1 with pT2N1 in endo group.	R1: 48% in Sx group and 14% in endo group	Not applicable

Supplementary Tables, di Pietro et al, National Institute for Health and Care Excellence (NICE) guidance on monitoring and management of Barrett's oesophagus and stage I oesophageal adenocarcinoma

Study (n)	Intervention	Comparator	Inclusion/exclusion criteria	Stage	Grade of differentiation	LVI	R0/R1	Submucosal grades
		ablative techniques or radical mucosectomy depending on the length of the Barrett's oesophagus. Photodynamic therapy was utilized to 2006 with radiofrequency ablation subsequently being the most common ablative therapy. APC was selectively applied.						
Zehetner, 2011<br>N=66 (in review analysis, at stage T1a)	Oesophagectomy was performed as a transthoracic en bloc, transhiatal, minimally invasive, or vagus-sparing resection. Reconstruction in all cases was with a	EMR. Occasionally, argon plasma coagulation was used to touch up small areas, typically at the time of endoscopic resection of a	Inclusion: All patients with high-grade dysplasia or intramucosal adenocarcinoma treated endoscopically or by an esophagectomy<br>Exclusion: Patients with tumors invasive into the submucosa were excluded, but lymphovascular invasion	In overall paper there were 21% in Sx group at stage T0 and 55% in endo group at stage T0. <u>However, it was possible to extract data separately for T1a</u>	No data	1 lymph node met in Sx group	R0: 100%	Not applicable

Supplementary Tables, di Pietro et al, National Institute for Health and Care Excellence (NICE) guidance on monitoring and management of Barrett's oesophagus and stage I oesophageal adenocarcinoma

Study (n)	Intervention	Comparator	Inclusion/exclusion criteria	Stage	Grade of differentiation	LVI	R0/R1	Submucosal grades
	tubularized gastric pull-up.	lesion. In some patients immediately after endoscopic resection of a nodule, the surrounding Barrett's mucosa was ablated with the Halo 90 device. Ablation was not performed over areas just resected.	or poor differentiation in an intramucosal lesion did not deter endoscopic therapy	stage by analysis of the text. Therefore, only T1a patients were included in the analyses for this review.				

Supplementary Tables, di Pietro et al, National Institute for Health and Care Excellence (NICE) guidance on monitoring and management of Barrett's oesophagus and stage I oesophageal adenocarcinoma

Supplementary Table 11: Summary of studies included in the evidence review for anti-reflux surgery to reduce disease progression

Study	Intervention and comparison	Population	Outcomes	Comments
Attwood 2008<br>(LOTUS trial)	Laparoscopic Nissen fundoplication (N=32)<br><br>Vs<br><br>Medical treatment with esomeprazole 20 mg od for their disease (N=28)	Adults aged 18–70 years with confirmed GERD, with or without Barrett's oesophagus.<br><br>Only results relevant to people with Barrett's oesophagus (N=60) have been extracted in the present review.<br><br>Mean age: 47 years (Surgical arm) and 50 years (Medical arm)<br><br>Europe	Treatment failure (Protocol outcome: adverse events)<br><br>During a 3-year follow-up	Long-Term Usage of Acid Suppression Versus Anti-Reflux Surgery trial (LARS)
Parrilla 2003	Anti-reflux surgery<br>(N=58) short Nissen fundoplication (1.5–3 cm) over a 48 to 50 French bougie through a laparotomy.<br>Vs<br>Medical treatment for all patients (N= 43) consisted of hygiene, diet, and postural measures as well as antisecretory drugs: H2 antagonists (150 mg twice daily) initially and omeprazole (20 mg twice daily) from 1992 onward for all patients.	Patients diagnosed with Barrett's oesophagus (including short Barrett's segment with intestinal metaplasia) (N=101)<br><br>Median age(range): Surgical arm: 43 years (10–71)<br>Medical arm: 50 years (12–78)<br>Spain	Progression to high-grade dysplasia<br><br>Dysplasia de novo (Progression to any grade dysplasia from non-dysplastic Barrett's oesophagus<br><br>Complications (Splenectomy. inability to belch or vomit, and mild and transitory postoperative dysphagia)<br><br>Median (range) 5 years follow-up (range 1–18)	N=91 had intestinal metaplasia; N=8 had low-grade dysplasia.<br><br>Low grade dysplasia:<br>Medical treatment: n=3 (7%)<br>Anti-reflux surgery: n=5 (9%)<br><br>Intestinal metaplasia:<br>Medical treatment: n=39 (91%)<br>Anti-reflux surgery: n= 52 (90%)<br>Downgraded for indirectness as children are included in the study participants.

Supplementary Tables, di Pietro et al, National Institute for Health and Care Excellence (NICE) guidance on monitoring and management of Barrett's oesophagus and stage I oesophageal adenocarcinoma
